# Whole-transcriptome sequencing identifies key differentially expressed circRNAs/lncRNAs/miRNAs/mRNAs and linked ceRNA networks in adult degenerative scoliosis

**DOI:** 10.3389/fnmol.2023.1038816

**Published:** 2023-03-30

**Authors:** Xin Shi, Panpan Li, Xiang Wu, Jun Shu

**Affiliations:** ^1^The Second Affiliated Hospital of Kunming Medical University, Kunming Medical University, Kunming, China; ^2^Faculty of Medicine and University Hospital of Cologne, University of Cologne, Cologne, Germany

**Keywords:** adult degenerative scoliosis, intervertebral disc degeneration, circRNAs/lncRNAs/miRNAs/mRNAs, whole blood, nucleus pulposus tissue

## Abstract

**Background:**

Adult degenerative scoliosis (ADS) is forecast to be a prevalent disabling condition in an aging society. Universally, its pathogenesis is perceived as intervertebral disc degeneration (IDD), however, a thought-provoking issue is why precisely a subset of patients with disc degeneration develop ADS. Exploring the diversities between common IDD and ADS would contribute to unraveling the etiological mechanisms of ADS. Therefore, we aimed to integrate the circRNA, lncRNA, miRNA, and mRNA expression profiles from normal adults (Normal), patients with lumbar disc herniation (LDH), and ADS by whole transcriptome sequencing, which identifies critical functional ncRNA and ceRNA networks and crosstalk between the various transcripts.

**Methods:**

The fresh whole blood samples (*n* = 3/group) were collected from ADS patients, LDH patients, and healthy volunteers (Normal group), which were examined for mRNA, miRNA, lncRNA, and circRNA expression and screened for differentially expressed (DE) ncRNAs. Then, Gene Ontology (GO) and KEGG analyses were performed for gene annotation and enrichment pathways on the DE RNAs, which were constructed as a lncRNA-miRNA-mRNA network. Eventually, DE RNAs were validated by qRT-PCR targeting disc nucleus pulposus (NP) tissue in ADS and LDH group (*n* = 10/group).

**Results:**

Compared to the LDH group, we identified 3322 DE mRNAs, 221 DE lncRNAs, 20 DE miRNAs, and 15 DE circRNAs in the ADS. In contrast to Normal, 21 miRNAs and 19 circRNAs were differentially expressed in the ADS. The expression of multiple differentially expressed ncRNAs was confirmed by qRT-PCR analysis to be consistent with the sequencing results. In addition, GO, and KEGG analysis demonstrated that most DE mRNAs and ncRNAs target genes are involved in various biological processes, including Endocytosis, Apoptosis, Rap1 signaling pathway, Notch signaling pathway, and others. The constructed lncRNA-miRNA-mRNA co-expression network was primarily related to angiogenesis and regulation.

**Conclusion:**

By focusing on comparing asymmetric and symmetric disc degeneration, whole-transcriptome sequencing and bioinformatics analysis systematically screened for key ncRNAs in the development of ADS, which provided an abundance of valuable candidates for the elucidation of regulatory mechanisms. The DE ncRNAs and the lncRNA-miRNA-mRNA network are intrinsically involved in the regulation of mediator and angiogenesis, which may contribute to the insight into the pathogenesis of ADS.

## Introduction

Adult degenerative scoliosis (ADS), attributed to degenerative changes without pre-existing spinal deformities, is characterized by a Cobb angle >10 degrees as measured in the coronal plane. The prevalence of vertebral deformity has ranged from 32% to 68% in the population over 65 years of age (Schwab et al., 2005). Globally, the prevalence of ADS is increasing at an unprecedented rate, contributed by advances in healthcare translating into longer life expectancy and acceleration of age-related spinal degeneration (Ploumis et al., 2007). ADS is a potentially self-limiting disease that would affect the heterogeneous community of patients. It is widely considered a consequence of asymmetric disc degeneration, osteoporosis, and vertebral compression fractures, which typically occur in the lumbar spine (Herkowitz and Kurz, 1991). Although, in terms of pathogenesis, ADS has the same starting point as degenerative spinal disease, usually relatively symmetrical intervertebral disc degeneration (IDD; as it occurs in the general population) cannot cause deformity. Lumbar disc herniation (LDH) is commonly associated with IDD. In contrast, in patients who ultimately develop ADS, which is typically characterized by asymmetry in the degeneration of multiple discs and small joints leading to the asymmetric collapse of the disc space, triggering progressive imbalance in axial loading and subsequent deformity (Benner and Ehni, 1979). Studies have shown that the application of stress leads to degenerative disc changes; however, it is unclear whether *in vivo* degeneration is due to the application of stress to discs that have undergone biochemical changes or to the response of normal discs to stress, which remains elusive in terms of the exact cellular and molecular mechanisms. Therefore, comparing the differences between asymmetric disc degeneration and symmetric disc degeneration would contribute to unraveling the pathogenesis of ADS. Notably, numerous genetic factors associated with potential regulatory mechanisms are dysregulated in IDD, particularly non-coding RNA (ncRNA; Adams and Roughley, 2006; Mayer et al., 2013; Zhu et al., 2019).

ncRNAs, the critical regulatory elements of genomic encoding, include microRNAs (miRNAs), long-stranded non-coding RNAs (lncRNAs), and the recently discovered circular RNAs (circRNAs), which play vital roles in a variety of cellular processes (Beermann et al., 2016). Different classes of ncRNAs have unique functions (Karali and Banfi, 2019). miRNAs serve as critical regulators of various physiological and pathological processes, including development, organogenesis, apoptosis, and cell proliferation and differentiation (Correia de Sousa et al., 2019). Meanwhile, miRNAs can regulate gene expression by degrading mRNAs with corresponding miRNA response elements (MREs; Reh and Hindges, 2018). CircRNAs, a class of endogenous ncRNAs widely expressed in eukaryotic cells, are characterized by a covalently closed loop structure without 5′ and 3′ polarity and poly-A tail structure (Chen et al., 2019), which ensures their high stability (Rong et al., 2017; Patop et al., 2019). CircRNA dysregulation is responsible for a variety of diseases, such as cancer, diabetes, cardiovascular disease, certain immune disorders, and neurodegenerative diseases (Tay et al., 2014; Kristensen et al., 2019). Moreover, circRNAs act as miRNA sponges to influence the function of miRNAs (Haque and Harries, 2017), and play a significant role in regulating gene expression by combining with specific microRNA or RNA-binding proteins to alleviate target mRNA degradation in a variety of cellular biological processes and diseases. The dysregulation of ncRNAs has been widely demonstrated in different types of diseases, including cancer, neurodegeneration, inflammatory/immune diseases, and orthopedic diseases such as spinal scoliosis, osteoporosis, osteoarthritis (Akhter, 2018; Chen et al., 2018, 2019; Shi et al., 2022). Next-generation sequencing contributes to understanding differentially expressed RNAs and their biological functions in ADS. Growing studies have shown that the expression profiles are different for ncRNA, including miRNA, circRNA, and lncRNA, between blood tissues obtained from healthy individuals and patients with LDH (Cui et al., 2020; Guo et al., 2020; Hiyama et al., 2022).

In previous studies, we have proposed a significant contribution of ncRNA in ADS, which revealed differential expression of lncRNAs and mRNAs between ADS and normal adults (Shi et al., 2022). Nevertheless, to date, lack of complete expression profiles integrating circRNA, lncRNA, miRNA, and mRNA and sequential inertia analysis of the dynamics of ncRNA differential expression between the non-degenerated disc, LDH, and ADS. In addition, the crosstalk of multiple ncRNAs in the ADS remains uncertain. Therefore, we collected whole blood (WB) tissue from healthy adult volunteers, patients with LDH, and ADS for whole transcriptome sequencing and validation in disc tissue, which comprehensively characterized the expression profiles of circRNA, lncRNA, miRNA, and mRNA. Furthermore, using various bioinformatics tools, we have systematically identified key circRNAs, lncRNAs, and miRNAs associated with the development of ADS and have partially unraveled the underlying regulatory networks.

## Materials and methods

### Sample collection

The study protocol was approved by the Medical Ethics Committee of Kunming Medical University. In accordance with the Declaration of Helsinki, written informed consent was obtained from all participants prior to enrolment.

Ten patients with ADS and 10 with LDH were recruited into the ADS and LDH groups, respectively. Meanwhile, 10 healthy volunteers from patients without disc degeneration who underwent lumbar spine surgery due to trauma were recruited into the normal group. All participants complied with the inclusion and exclusion criteria, as shown in Tables 1, 2.

**Table 1:** The inclusion criteria.

The inclusion criteria of each group were as follows
(1) Age: Normal group: 18–35 years old; LDH group & ADS group: Over 50 years old
(2) 18.5 ≤ Body Mass Index (BMI) < 24
(3) Diagnose:
Normal group: After X-ray, CT and MRI examination, there is no spine-related disease, intervertebral disc and facet joint structure is complete, and there is no lesion
LDH group: CT and MRI suggested different degrees of degeneration of intervertebral discs, intervertebral facet joints, ligamentum flavum, etc. All patients were diagnosed with lumbar disc herniation by radiograph, CT and MRI
ADS group: According to the medical history, clinical examination and imaging examination were diagnosed as ADS; With the lumbar spine as the vertex, X-ray showed that Cobb Angle range of scoliosis on the coronal plane was greater than or equal to 15°

**Table 2:** The exclusion criteria.

The exclusion criteria of each group were as follows
(1) Medical history of autoimmune diseases, systemic inflammatory diseases, solid tumors or hematological malignancies
(2) Complicated with severe osteoporosis, severe liver and kidney dysfunction
(3) Pregnant or lactating women
(4) Medical history of metabolic bone disease, spinal trauma, spinal infection
(5) History of spinal surgery
(6) Scoliosis secondary to other organic spinal lesions, such as tumor, trauma, tuberculosis, metabolism, etc.
(7) A history of lumbar spine surgery, congenital scoliosis, or undetected idiopathic spinal column scoliosis in adolescents

**Figure 1:**
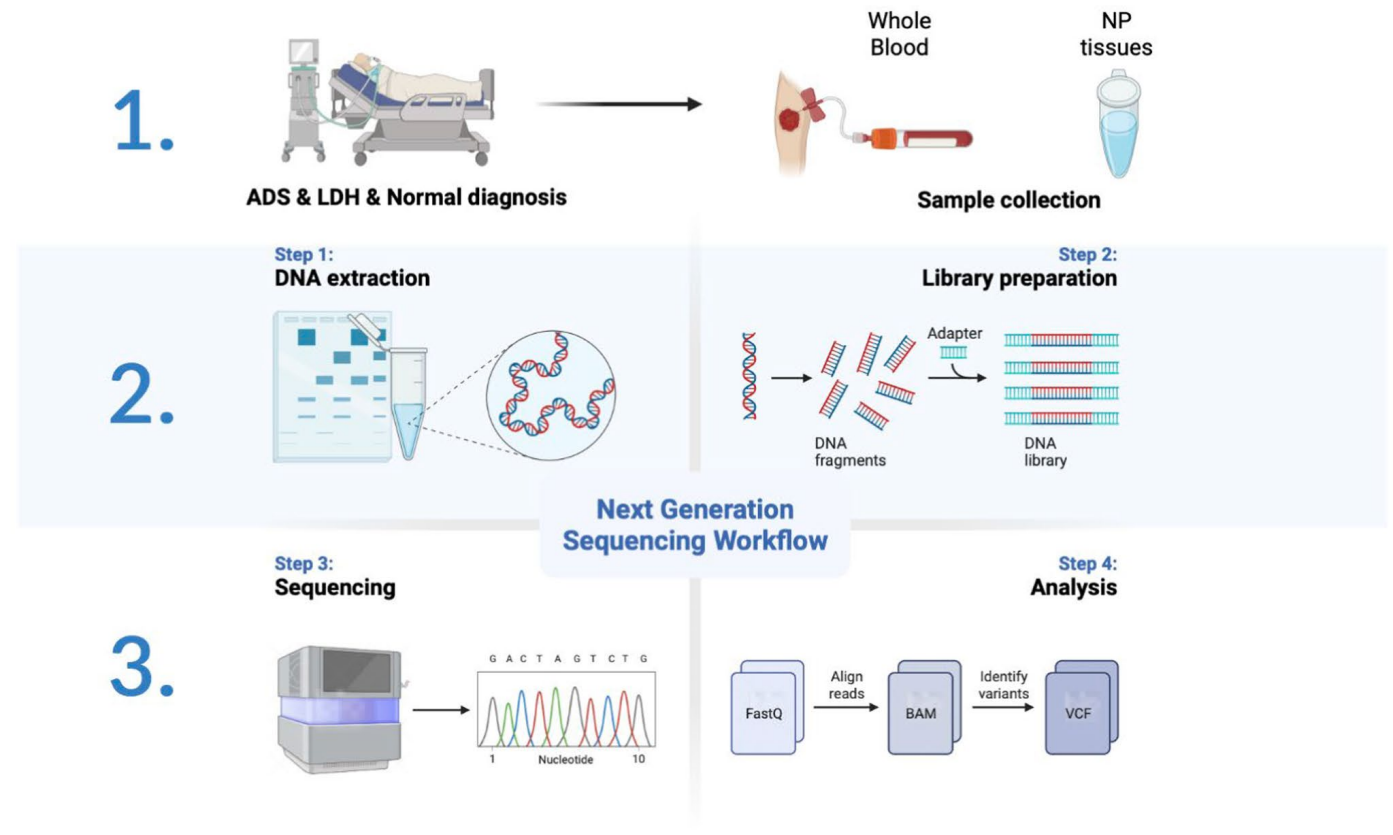
Schematic diagram of the workflow of this study.

All participants were collected with WB samples pre-operatively and NP tissue from the intervertebral disc intra-operatively. Pre-operation, a fasting WB sample (5 mL × 2/participant) was obtained from the middle vein of each participant’s elbow between 9:00 a.m. and 9:30 a.m. For all WB samples, incubate in PAXgene Blood RNA tubes (BD, United States) at −20°C for 24 h and then transfer to −80°C for preservation in a refrigerator. After all, samples have been collected, the tubes of WB RNA are thawed at room temperature (18°C–25°C) for 2 h on a metal rack. Once completely thawed, carefully invert the WB RNA tube 10 times. Finally, nine samples were subjected to total RNA extraction, detection, lncRNA library construction, and sequencing (Figure 1).

### Total RNA extraction

For RNA-seq, total RNA was extracted from each sample using Trizol reagent (Invitrogen, Carlsbad, CA, United States) according to the manufacturer’s protocol. Then, the concentration and quality of total RNA were assessed by applying a NanoDrop ND 1000 spectrophotometer (NanoDrop Technologies, Wilmington, DE, United States). Simultaneously, the integrity of the total RNA was determined by an Agilent 2,100 Bioanalyzer (Agilent Technologies, Santa Clara, CA, United States) and kept at −80°C. Only high-quality RNA samples (RNA integrity number > 7.0) were selected for cDNA library construction.

### cDNA library construction and sequencing

A total of nine WB samples (three ADS, three LDH, and three normal) were subjected to whole transcriptome analysis employing high-throughput sequencing. For mRNA, lncRNA, and circRNA sequencing, ribosomal RNA (rRNA) was removed from total RNA (1 μg) by the Ribo-Zero Gold rRNA kit (Illumina, San Diego, CA, United States) and sequenced using a strand-specific cDNA library. According to the manufacturer’s instructions, the TrueSeq RNA sample prep Kit (Illumina) is applied for preparation without purification and enrichment of mRNA. For miRNA sequencing, the TrueSeq Small RNA Library Prep Kit (Illumina) is employed to prepare micro RNA in accordance with the manufacturer’s recommended protocol. Briefly, the RNA is ligated to T4 RNA Ligase 1 at the 3′ end and T4 RNA Ligase 2 at the 5′ junction and then reverse transcribed into cDNA. Following PCR amplification, the products were purified by polyacrylamide gel electrophoresis. All libraries generated were validated by Agilent Bioanalyzer 2200 system (Agilent Technologies) and then sequenced *via* the HiSeq™ 2000 (Illumina) platform.

After that, the raw sequence of the FASTQ format was trimmed using Trimmomatic to remove splices and low-quality reads, on which the clean reads were aligned to the hg19 human reference genome by using Hisat2. A StringTie was employed to assemble the aligned reads into the known transcripts in each sample. Finally, all samples were amalgamated to re-construct a comprehensive transcriptome. Additionally, the Cuffcompare program is intended to elucidate the genomic position of transcripts by comparing the integrated transcripts with the annotated transcripts. Meanwhile, transcripts with codes “I,” “U,” “X,” and “O” and transcripts longer than 200 bp that contain two or more exons have been retained. Four analysis programs, including the coding-noncoding index (CNCI), the coding potential calculator (CPC), a lncRNA and mRNA predictor based on the modified k-mer scheme (PLEK), and the protein family database (Pfam), were applied to predict the coding capacity of transcripts. For lncRNA analysis, in which transcripts with coding potential are filtered out.

Furthermore, CIRI (V2.0) software was used to identify circRNAs. Briefly, the Burrows-Wheeler Alignment (BWA) mapping tool compares clean reads versus the human reference genome hg19 to obtain a sequence alignment map (SAM) file, where balanced ligated reads with paired cross-shear signals are detected. Then, ligated reads were filtered by double-end mapping (PEM) and GT-AG splice signals, while ligated reads were detected by the DM algorithm.

In terms of miRNA analysis, raw sequencing reads were trimmed by Cutadapt to identify and remove splice fragments, while sequences <15 bp or >41 bp in length were filtered. Also, clean reads were obtained by removing low-quality (>80% of Q20) reads with the FASTX-Toolkit software and excluding reads with poly N using the NGS QC toolkit.

Clean reads were searched against the Rfam database^1^ to exclude other ncRNAs, including rRNA, tRNA, snRNA, and snoRNA, with the aid of RepeatMasker.^2^ Putative known miRNAs were identified by searching the miRBase 21.0 database,^3^ while potential novel miRNAs were predicted from the remaining unannotated reads by miRDeep2 software.

### Analysis of differential expression

The datasets generated from the pre-sequencing study (GSE209825), which were co-incidentally analyzed for differential expression. The expression levels of mRNAs and lncRNAs were determined by calculating Fragments Per Kilobase of transcript per Million fragments mapped (FPKM). The expression levels of mRNAs and lncRNAs were determined by calculating Fragments Per Kilobase of transcript per Million fragments mapped (FPKM). Further, the DESeq R software package was used to assess the differential expression of mRNA, lncRNA, miRNA, and circRNA that conformed to the threshold value of *p* < 0.05 and fold change >1. Eventually, miRNA, mRNA, lncRNA, and circRNA that adhered to |log2 fold change| ≥ 1 and *p* < 0.05 were screened, depending on the significance analysis and *p*-values.

To outline the differential expression profiles of the transcripts, generating volcano maps *via* the R ggplot2 package and performing hierarchical cluster analysis *via* the Manhattan distance metric and the Ward minimum variance method from the heatmap package in R.

### Gene ontology and Kyoto encyclopedia of genes and genomes pathway analysis

Gene Ontology (GO)^4^ analysis is based on three annotated ontologies, including the exploration of molecular function (MF), cellular components (CC), and biological processes (BP), which originate from differentially expressed (DE) mRNAs, DE lncRNAs and miRNAs targeting genes, and the gene function of DE circRNAs host genes. Simultaneously, Kyoto encyclopedia of genes and genomes (KEGG)^5^ analysis was performed to assess the biological functions and enrichment pathways of the DE genes. GO, and KEGG analyses were performed by assigning R packages based on hypergeometric distributions.

### Real-time fluorescence quantitative PCR analysis

In total, 20 NP tissue samples (ADS group, LDH group, *n* = 10/group) were collected to validate DE RNAs by real-time PCR analysis. The criteria for selecting RNAs to perform RT-PCR were based on the level of expression in the whole transcriptome analysis, significant differences, or supervision by functional analysis. In order to quantify mRNA and lncRNA levels, 500 ng of the whole RNA was reverse transcribed to synthesize cDNA, which was accomplished using the TransScript^®^ All-in-One First-Strand cDNA Synthesis SuperMix Kit with gDNA remover (TransGen Biotech, Beijing, China). Following the manufacturer’s instructions, first-strand cDNA synthesis was carried out by reverse transcription with TransScript^®^ to quantify miRNA levels (*Trans* PCR SuperMix,TransGen Biotech). Real-time PCR reactions were performed on the LightCycler^®^ 480 II (Roche, Basel, Switzerland) employing a PerfectStart™ Green qPCR SuperMix (TransGen Biotech). In addition, GAPDH was applied as an internal control for the normalization of mRNA and lncRNA levels, while miRNA levels were normalized with U6. Specific primer sequences are presented in Table 3. The 2^−ΔΔCt^ method was employed to calculate the relative RNA expression level. This value is represented as mean ± SD. A student *t*-test was conducted, and when *p*-value < 0.05, the results were considered to be significantly different.

**Table 3:** Primers used for qRT-PCR analysis of DE mRNA and ncRNAs levels.

Name	Primer sequence
*TPT1*	F: 5′-TGAAGAACAGAGACCAGAAAG-3′ R: 5′-CACGGTAGTCCA ATAGAGCAAC-3′
*RIOK3*	F: 5′-TGT GGC ATG CTG GAA AGG TCT G-3′ R: 5′-GCT TCC TTG ACT CCT CCT TTC TGG-3′
*ASAP1*	F: 5′-TG TAGTCTTACTTGAAGAGGATGGACC-3′ R: 5′-CCCTC CCAGCCCACTACCT-3′
*SEC14L1*	F: 5′-CTGCTACACCGTTCACCCTGA-3′ R: 5′-GGGGCACAAAGGTTATGCCTT-3′
*XLOC_000209*	F: 5′-TGAGATAGTTTCAAACTGCT-3′ R: 5′-AGTCTTTTGATCTACTACAC-3′
*hsa-miR-224-5p*	F: 5′-CTGGTAGGTAAGTCACTA-3′ R: 5′-TCAACTGGTGTCGTGGAG-3′
*hsa-miR-133a-3p*	F: 5′-UUU GGU CCC CUU CAA CCA GCU G-3′ R: 5′-UAA ACC AAG GUA AAA UGG UCG A-3′
*hsa_circ_0019079*	F: 5′-TGAAATGGAGGAGATCTAAATGT-3′ R: 5′-GCCAGCCTTTCAACTTCCTC-3′
*hsa_circ_0000179*	F: 5′-CTAGCAGTTGCCAATGAAGAAG-3′ R: 5′-ATTGATGACCTTTGCATGTTCC-3′

### Competitive endogenous RNA networks

Universally, RNA transcripts with miRNA binding sites (circRNAs, lncRNAs, and mRNAs) could compete and mutually influence the same miRNA, which species are referred to as competing endogenous RNAs. The binding of targets and miRNAs could be predicted by TargetScan^6^ and miRanda.^7^ Moreover, a potential lncRNA-miRNA-mRNA network was constructed through the competing endogenous RNA hypothesis. The above data were visualized by Cytoscape software (version 3.7.2) to investigate the contribution of lncRNA -miRNA-mRNA ceRNA networks in the pathogenesis of ADS.

### Statistical analysis

Data are presented as the mean ± SD of the results derived from at least three independent experiments. Diagrams were constructed and statistically analyzed by GraphPad Prism 9 (GraphPad Software, La Jolla, CA, United States). Appropriately, the Student’s *t*-test and Mann–Whitney *U*-test were applied to determine significant differences between groups. The inter-sample correlation of expression was tested by applying the Pearson correlation coefficient. For all tests, *p*-values < 0.05 were considered statistically significant. In addition, as a correction for batch effects, the RUVseq package for the R language was employed for batch correction. Alternatively, heat maps and volcano plots were exported from the R language heat map package 2, whereas scattering plots and principal component analysis results were exported from the ggplot2 package.

## Results

### Three-dimensional principal component analysis and hierarchical clustering analysis

**Figure 2:**
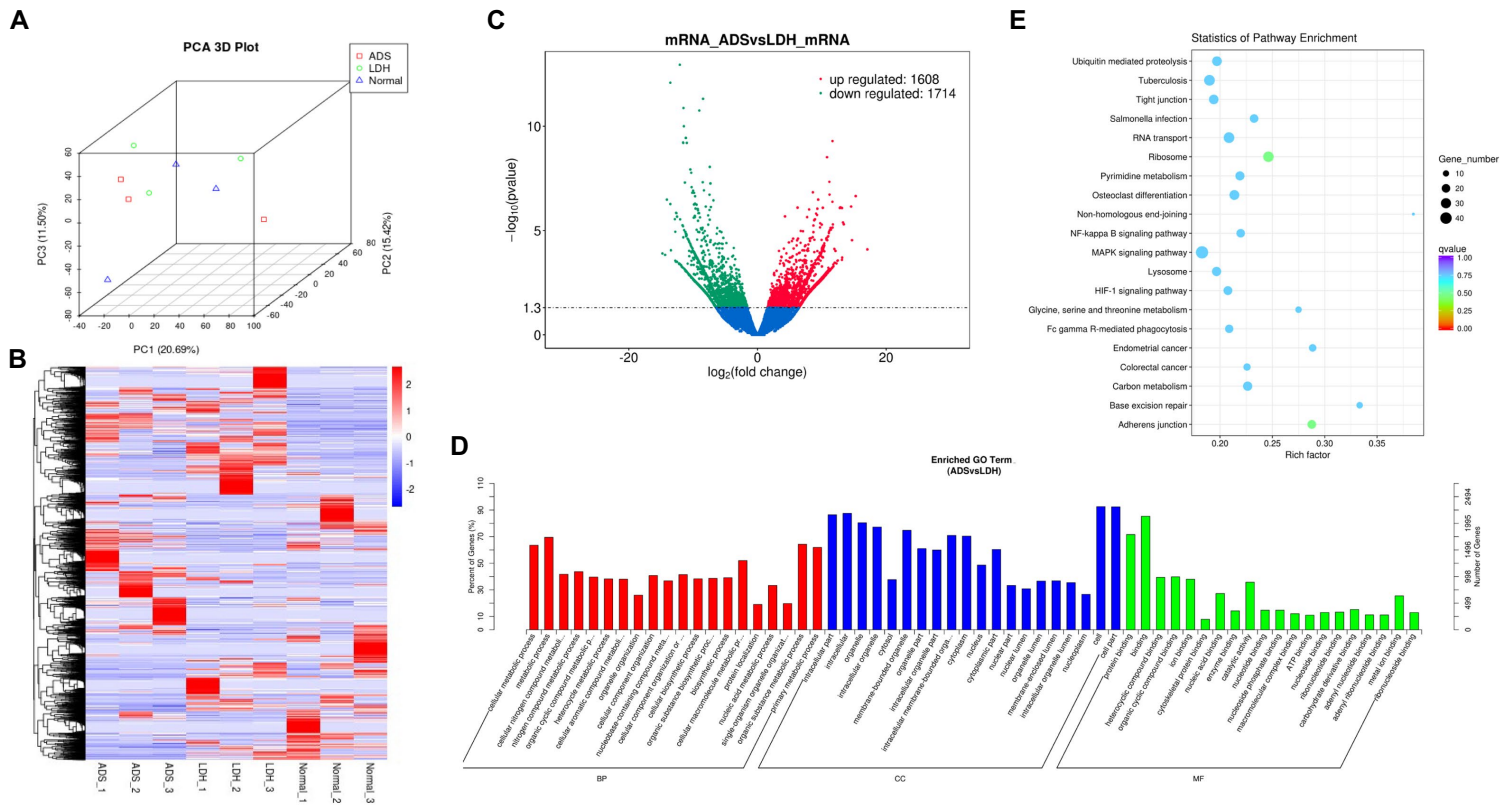
Identification of differentially expressed mRNAs in ADS **(A)** Principal component analysis shows the clustering of mRNA in different samples. **(B)** Hierarchical clustering illustrates distinguished expression difference of mRNA and between the three groups and homogeneity between groups. **(C)** Volcano plot showing differentially expressed mRNAs in ADS and LDH group. **(D)** The 20 most enriched Gene Ontology terms for the parental genes of the differentially expressed mRNAs. Enriched GO terms are on the vertical axis, and the number of annotated differentially expressed genes associated with each GO term is indicated on the horizontal axis. **(E)** The 20 most enriched KEGG pathways for the differentially expressed mRNAs. The size of the symbol represents the number of genes, and the colors represent *p*-value.

**Figure 3:**
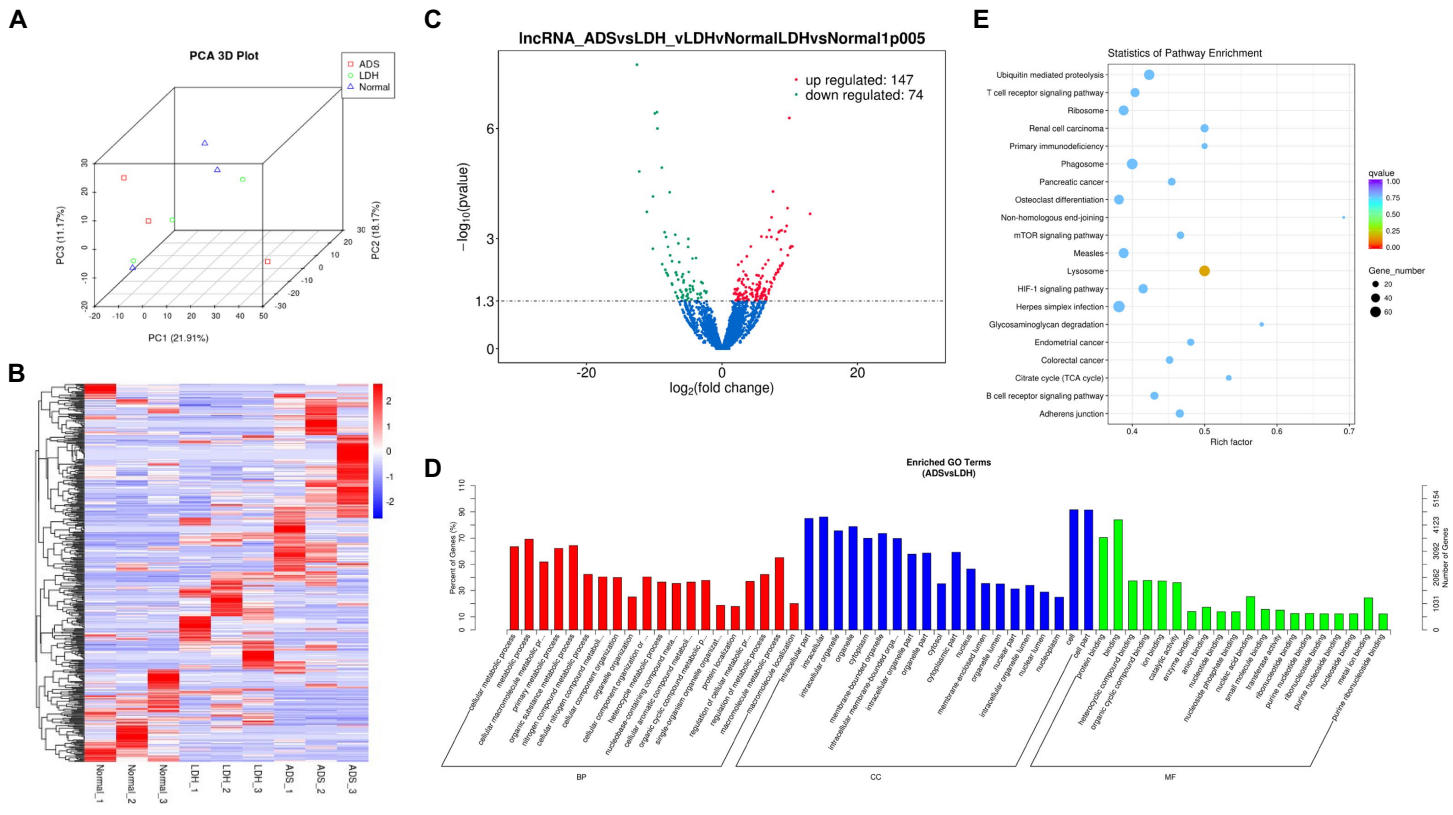
Identification of differentially expressed lncRNAs in ADS **(A)** Principal component analysis shows the clustering of lncRNA in different samples. **(B)** Hierarchical clustering illustrates distinguished expression difference of lncRNA and between the three groups and homogeneity between groups. **(C)** Volcano plot showing differentially expressed lncRNAs in ADS and LDH group. **(D)** The 20 most enriched Gene Ontology terms for the parental genes of the differentially expressed lncRNAs. Enriched GO terms are on the vertical axis, and the number of annotated differentially expressed genes associated with each GO term is indicated on the horizontal axis. **(E)** The 20 most enriched KEGG pathways for the differentially expressed lncRNAs. The size of the symbol represents the number of genes, and the colors represent *p*-value.

**Figure 4:**
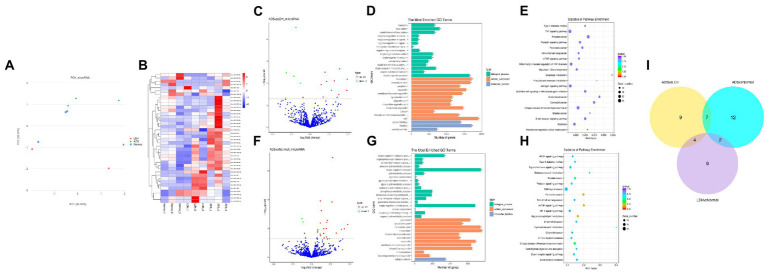
Identification of differentially expressed miRNAs in ADS **(A)** Principal component analysis shows the clustering of miRNA in different samples. **(B)** Hierarchical clustering illustrates distinguished expression differences of miRNA and between the three groups and homogeneity between groups. **(C)** Volcano plot showing differentially expressed miRNAs in ADS and LDH group. **(D)** The 20 most enriched Gene Ontology terms for the parental genes of the differentially expressed miRNAs in ADS and LDH group. **(E)** The 20 most enriched KEGG pathways for the differentially expressed miRNAs in ADS and LDH group. **(F)** Volcano plot showing differentially expressed miRNAs in ADS and Normal group. **(G)** The 20 most enriched Gene Ontology terms for the parental genes of the differentially expressed miRNAs in ADS and Normal group. **(H)** The 20 most enriched KEGG pathways for the differentially expressed miRNAs in ADS and Normal group. Enriched GO terms are on the vertical axis, and the number of annotated differentially expressed genes associated with each GO term is indicated on the horizontal axis. The size of the symbol represents the number of genes, and the colors represent *p*-value in KEGG. **(I)** The Venn diagram created by the differentially expressed data of miRNAs.

**Figure 5:**
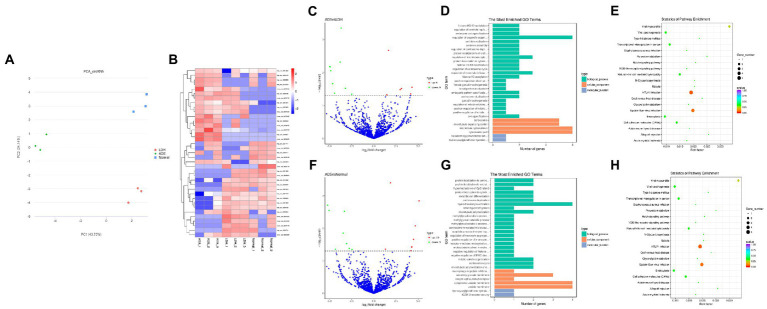
Identification of differentially expressed circRNAs in ADS **(A)** Principal component analysis shows the clustering of circRNA in different samples. **(B)** Hierarchical clustering illustrates distinguished expression differences of circRNA and between the three groups and homogeneity between groups. **(C)** Volcano plot showing differentially expressed circRNAs in ADS and LDH group. **(D)** The 20 most enriched Gene Ontology terms for the parental genes of the differentially expressed circRNAs in ADS and LDH group. **(E)** The 20 most enriched KEGG pathways for the differentially expressed circRNAs in ADS and LDH group. **(F)** Volcano plot showing differentially expressed circRNAs in ADS and Normal group. **(G)** The 20 most enriched Gene Ontology terms for the parental genes of the differentially expressed circRNAs in ADS and Normal group. **(H)** The 20 most enriched KEGG pathways for the differentially expressed circRNAs in ADS and Normal group. Enriched GO terms are on the vertical axis, and the number of annotated differentially expressed genes associated with each GO term is indicated on the horizontal axis. The size of the symbol represents the number of genes, and the colors represent *p*-value in KEGG.

Primarily, in order to determine whether there was clustering or outliers in the sample set, the differences between the clustering of the mRNA (Figures 2A,B), lncRNA (Figures 3A,B), miRNA (Figures 4A,B), and circRNA (Figures 5A,B) expression matrixes of the ADS, LDH, and Normal samples in different datasets were examined using PCA and hierarchical clustering analysis. The results indicated distinguishable ncRNAs expression profiles between different WB samples.

### Identification of differentially expressed mRNAs in ADS

DE mRNAs are displayed in the volcano plot (Figure 2C). In total, 3322 mRNAs were significantly differentially expressed in ADS tissues compared to LDH tissues, of which 1608 mRNAs were up-regulated, and 1714 mRNAs were down-regulated. The top 10 DE mRNAs are shown in Table 4. Notably, the top 10 most abundant GO terms in each category are shown in Figure 2D, which account for the metabolic process in the BP category, cell part in the CC category, and binding in the MF category. In addition, KEGG pathway analysis showed that DE mRNAs were enriched in several pathways, including Ribosome, Adheres junction, MAPK signaling pathway, and Tuberculosis (Figure 2E).

**Table 4:** The top 10 DE mRNAs.

mRNA_ID	Gene_ID	Gene_name	Log_2_(fold change)	Value of *p*	Regulated
ENST00000370857	ENSG00000076770	*MBNL3*	17.0854145	8.05E-05	Up
ENST00000577501	ENSG00000101782	*RIOK3*	15.2749598	2.23E-07	Up
ENST00000379060	ENSG00000133112	*TPT1*	14.6972161	2.90E-05	Up
ENST00000397857	ENSG00000160255	*ITGB2*	14.5635881	7.08E-07	Up
ENST00000646036	ENSG00000027697	*IFNGR1*	13.4168071	5.04E-06	Up
ENST00000361689	ENSG00000127603	*MACF1*	−14.768821	0.00012617	Down
ENST00000430767	ENSG00000129657	*SEC14L1*	−14.353966	0.00014747	Down
ENST00000518721	ENSG00000153317	*ASAP1*	−14.076165	3.31E-07	Down
ENST00000644218	ENSG00000166340	*TPP1*	−13.541962	8.19E-13	Down
ENST00000356338	ENSG00000197535	*MYO5A*	−13.536251	5.39E-07	Down

**Figure 6:**
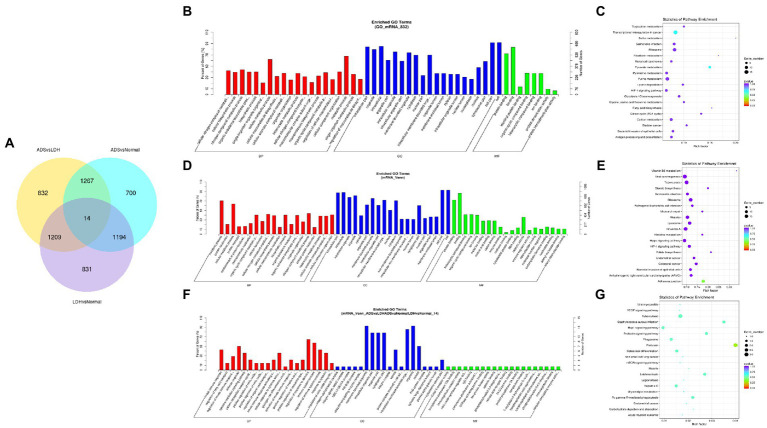
Identification of differentially expressed mRNAs combined with the GSE209825 dataset in ADS **(A)** The Venn diagram created by the differentially expressed data of mRNAs was combined with the GSE209825 dataset. **(B)** The 20 most enriched Gene Ontology terms for the parental genes of 832 significantly DE mRNAs in the ADS compared to the LDH group. **(C)** The 20 most enriched KEGG pathways for 832 significantly DE mRNAs. **(D)** The 20 most enriched Gene Ontology terms for the parental genes of 1,267 co-significantly DE mRNAs in the ADS compared to the LDH and Normal group. **(E)** The 20 most enriched KEGG pathways for 1,267 significantly DE mRNAs. **(F)** The 20 most enriched Gene Ontology terms for the parental genes of 14 co-significant DE mRNAs. **(G)** The 20 most enriched KEGG pathways for 14 co-significantly DE mRNAs. Enriched GO terms are on the vertical axis, and the number of annotated differentially expressed genes associated with each GO term is indicated on the horizontal axis. The size of the symbol represents the number of genes, and the colors represent *p*-value in KEGG.

A comprehensive analysis was conducted with the GSE209825 dataset, as illustrated in the Venn diagram (Figure 6A), a total of 832 significantly DE mRNAs were independently present in the ADS compared to the LDH group, whose GO terms are shown in Figure 6B: the most enriched GO terms consisted of metabolic process in the BP category, cell in the CC category and binding in the MF category. Moreover, KEGG pathway analysis revealed that DE mRNAs were enriched in several pathways, including Transcriptional misregulation in cancer, Pyruvate metabolism, Purine metabolism, and Ribosome (Figure 6C).

Nevertheless, when the ADS group was compared to both the LDH and Normal groups, 1267 co-significantly DE mRNAs were demonstrated in the ADS, which GO terms are illustrated in Figure 6D: the most enriched GO terms cover the metabolic process in the BP category, cell in the CC category, and binding in the MF category. In addition, the KEGG pathway Analysis revealed that DE mRNAs were enriched in several pathways, including Adheres junction, Viral carcinogenesis, Influenza A, and Hippo signaling pathway (Figure 6E). Interestingly, among the comparison group of the three pairs of differential expression analyses, a total of 14 co-significant DE mRNAs were identified, whose GO terms are shown in Figure 6F: the most abundant GO terms consisted of regulation of response to stimulus in the BP category, membrane-bounded in the CC category, and organelle in the CC category. Meanwhile, KEGG pathway analysis revealed that DE mRNAs were enriched in several pathways, including Pertussis, Tuberculosis, Rap1 signaling pathway, and Staphylococcus aureus infection (Figure 6G).

### Identification of differentially expressed lncRNAs in ADS

Compared to LDH tissues, a total of 221 significantly DE lncRNAs were detected in ADS, of which 147 lncRNAs were up-regulated, and 74 lncRNAs were down-regulated (The top 10 DE lncRNAs shown in Table 5). The DE lncRNAs are presented in the volcano plot (Figure 3C). Notably, by GO analysis of DE lncRNA target genes, the top 10 most enriched GO terms in each category are illustrated in Figure 3D, which comprise metabolic process in the BP category, cell part in the CC category, and binding in the MF category. In addition, KEGG pathway analysis revealed that DE mRNAs were enriched in several pathways, including Lysosome, Herpes simplex infection, Ubiquitin mediated proteolysis, and Ribosome (Figure 3E).

**Table 5:** The top 10 DE lncRNAs.

lncRNA ID	Gene_ID	Log_2_(fold change)	Value of *p*	Regulated
LNC_000009	XLOC_000209	13.0039315	0.00021117	Up
LNC_000024	XLOC_000209	10.4091033	0.00162821	Up
ENST00000440408.5	ENSG00000233864.7	10.1718082	0.00161752	Up
LNC_004416	XLOC_105578	10.0365058	0.00180891	Up
LNC_005442	XLOC_135387	9.92650142	5.16E-07	Up
LNC_000045	XLOC_000500	−12.552532	1.83E-08	Down
LNC_000017	XLOC_000209	−12.215847	1.49E-05	Down
LNC_003673	XLOC_086548	−11.087566	0.00018779	Down
LNC_000018	XLOC_000209	−10.210142	0.00189632	Down
ENST00000582008.5	ENSG00000263753.6	−10.192206	7.12E-05	Down

**Figure 7:**
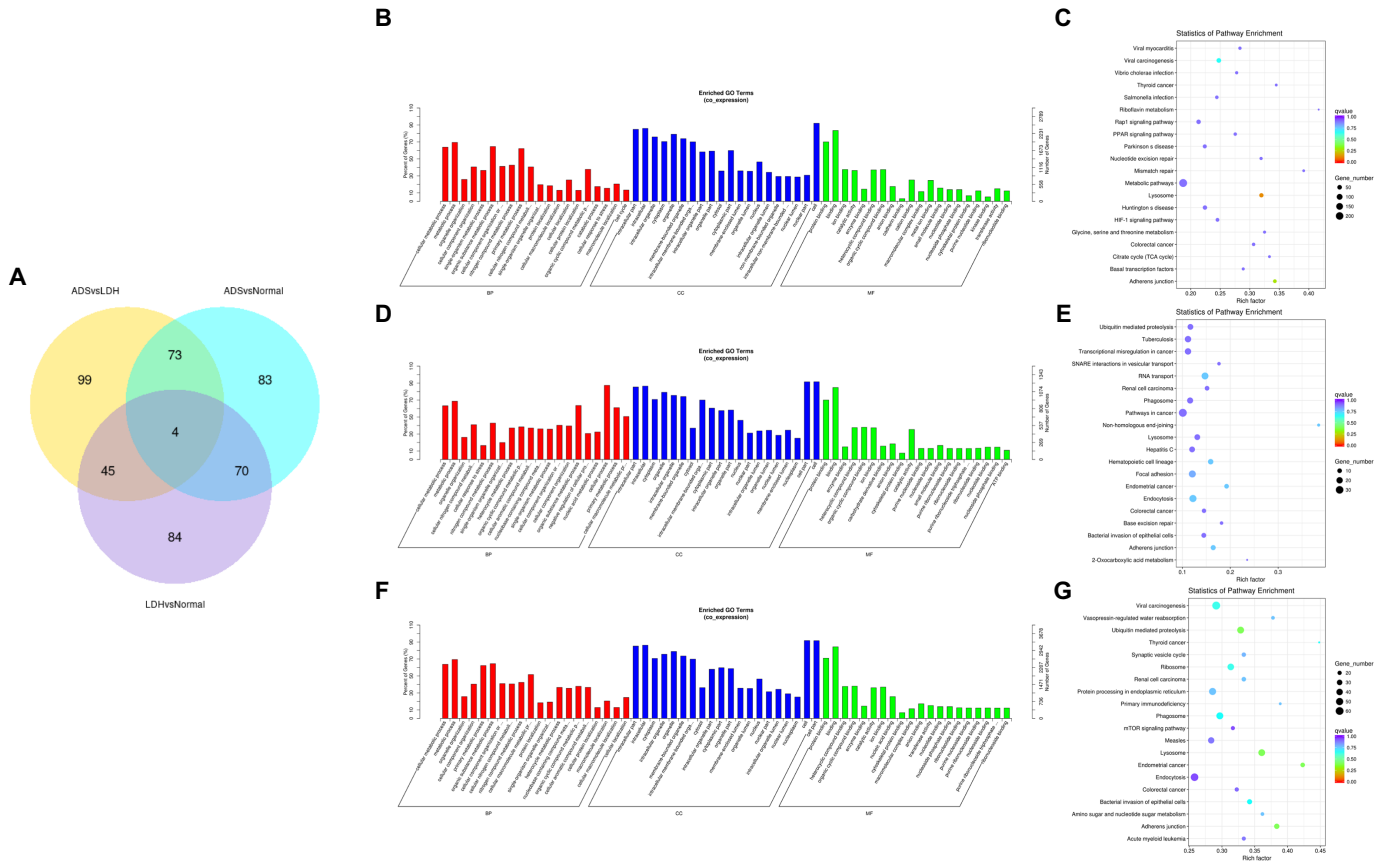
Identification of differentially expressed lncRNAs combined with the GSE209825 dataset in ADS **(A)** The Venn diagram created by the differentially expressed data of lncRNAs was combined with the GSE209825 dataset. **(B)** The 20 most enriched Gene Ontology terms for the parental genes of 73 co-significantly DE lncRNAs in the ADS compared to the LDH and Normal group. **(C)** The 20 most enriched KEGG pathways for 73 co-significantly DE lncRNAs. **(D)** The 20 most enriched Gene Ontology terms for the parental genes of 4 co-significant DE lncRNAs. **(E)** The 20 most enriched KEGG pathways for 4 co-significantly DE lncRNAs. **(F)** The 20 most enriched Gene Ontology terms for the parental genes of 99 significantly DE lncRNAs in the ADS compared to the LDH group. **(G)** The 20 most enriched KEGG pathways for 99 significantly DE lncRNAs. Enriched GO terms are on the vertical axis, and the number of annotated differentially expressed genes associated with each GO term is indicated on the horizontal axis. The size of the symbol represents the number of genes, and the colors represent *p*-value in KEGG.

Subsequently, in concordance with the GSE209825 data set, overlapping the results of the differential expression analysis of the three groups (Figure 7A). Compared to the LDH and Normal groups, 73 lncRNAs were significantly differentially expressed in the ADS, whose corresponding GO terms are shown in Figure 7B, among which the most enriched GO terms account for the metabolic process in the BP category, cell in the CC category and binding in the MF category. In addition, KEGG pathway analysis suggested that the DE lncRNAs are enriched in several pathways, including Lysosome, Adheres junction, Viral carcinogenesis, and Metabolic pathways (Figure 7C).

Interestingly, four lncRNAs were simultaneously present in three pairs of comparison groups for differential expression analysis, with associated GO terms illustrated in Figure 7D among which the most enriched GO terms contained cellular process in the BP category, cell in the CC category and binding in the MF category. In addition, KEGG pathway analysis demonstrated that the DE lncRNAs were enriched in several pathways, including Endocytosis, RNA transport, Hematopoietic cell lineage, and Endometrial cancer (Figure 7E).

In particular, 99 lncRNAs were present exclusively compared to the LDH group, with GO terms as presented in Figure 7F: the most enriched GO terms contained metabolic process in the BP category, cell in the CC category, and binding in the MF category. Additionally, KEGG pathway analysis indicates that DE lncRNAs are enriched in several pathways, including Ubiquitin mediated proteolysis, Lysosome, Adheres junction, and Endometrial cancer (Figure 7G).

### Identification of differentially expressed miRNAs in ADS

Compared to LDH, a total of 20 miRNAs were significantly differentially expressed in ADS, among which 13 miRNAs were up-regulated, and seven miRNAs were down-regulated (top 10 DE miRNAs shown in Table 6), as visualized in the volcano plot (Figure 4C). To further elucidate the biological functions of the DE miRNAs, the miRanda software was employed to predict the potential target genes. Overall, a total of 2,225 putative target genes, derived from 20 DE miRNAs, were identified and retained for GO and KEGG pathway analyses. The top 10 most enriched GO terms in each category related to the single-organism process in the BP category, cell in the CC category, and binding in the MF category (Figure 4D). Additionally, KEGG pathway analysis revealed that DE circRNAs were enriched in several pathways, including the TNF signaling pathway, Inflammatory mediator regulation of TRP channels, Estrogen signaling pathway, and the KEGG pathway (Figure 4E).

**Table 6:** The top 10 DE miRNAs.

miRNA name	Log_2_(fold change)	Value of *p*	Regulated
hsa-miR-224-5p	7.3074	0.020478	Up
hsa-miR-34c-5p	4.9356	0.0065943	Up
hsa-miR-4,707-3p	4.8635	0.021316	Up
hsa-miR-375-3p	3.9169	0.0022288	Up
hsa-miR-34a-5p	3.8073	0.032184	Up
hsa-miR-196a-5p	−3.2423	0.046554	Down
hsa-miR-133a-3p	−3.1474	0.0033334	Down
hsa-miR-499a-5p	−2.1497	0.0048173	Down
hsa-miR-499b-3p	−2.1497	0.0048173	Down
hsa-miR-122-5p	−1.8935	2.94E-05	Down

In addition, compared to the Normal group, 21 miRNAs were significantly differentially expressed in the ADS, among which 19 miRNAs were up-regulated, and two were down-regulated (Figure 4F). A total of 963 putative target genes for the 21 DE miRNAs were analyzed for GO and KEGG pathways. Further, the most enriched GO terms refer to the single-organism process in the BP category, intracellular in the CC category, and catalytic activity in the MF category (Figure 4G). Additionally, KEGG pathway analysis indicates that DE miRNAs are enriched in several pathways, including the mTOR signaling pathway, Glycerophospholipid metabolism, and Pancreatic cancer (Figure 4H).

Interestingly, compared to the LDH and Normal groups, the Venn diagram revealed that seven miRNAs were significantly differentially co-expressed in the ADS. In contrast, nine miRNAs were exclusively present when compared to LDH (Figure 4I).

### Identification of differentially expressed circRNAs in ADS

Compared to the LDH group, a total of 15 circRNAs were significantly differentially expressed in the ADS group (fold change > 1, *p* < 0.05), among which six circRNAs were up-regulated, and nine circRNAs were down-regulated (Top 10 DE circRNAs shown in Table 7), as shown in the volcano plot for DE circRNAs (Figure 5C).To predict the potential function of the DE circRNAs, which correspond to the host genes were subjected to GO and KEGG pathway analysis. The top 10 most enriched GO terms in each category are illustrated in Figure 5D, which encompasses regulation of organelle organization in the BP category, cytoskeletal part in the CC category, and transforming growth factor beta receptor, common-partner cytoplasmic mediator activity in the MF category. In addition, KEGG pathway analysis demonstrated that DE circRNAs were enriched in several pathways, including HTLV-I infection, Epstein–Barr virus infection, Viral myocarditis, and Viral carcinogenesis (Figure 5E).

**Table 7:** The top 10 DE circRNAs.

circRNA name	Source_gene_ID	Source_gene_name	Log_2_(fold change)	Value of *p*	Regulated
hsa_circ_0006396	ENSG00000089234	*BRAP*	4.2935	0.027883	Up
hsa_circ_0019079	ENSG00000138182	*KIF20B*	4.1499	0.044547	Up
hsa_circ_0000179	ENSG00000143476	*DTL*	3.1129	0.030503	Up
hsa_circ_0002319	ENSG00000186001	*LRCH3*	2.9651	0.032859	Up
novel_circ_0001726	ENSG00000063854	*HAGH*	1.6522	0.0021132	Up
novel_circ_0002306	ENSG00000141646	*SMAD4*	−4.9607	0.0065715	Down
hsa_circ_0067619	ENSG00000114127	*XRN1*	−4.9036	0.0066811	Down
novel_circ_0004714	ENSG00000138756	*BMP2K*	−4.8541	0.0087296	Down
novel_circ_0004327	ENSG00000114166	*KAT2B*	−4.626	0.021594	Down
hsa_circ_0005734	ENSG00000189319; ENSG00000258539	*FAM53B;* *AC068896.1*	−4.4091	0.04205	Down

Moreover, compared to the Normal group, 19 circRNAs were significantly differentially expressed in the ADS group, of which 10 circRNAs were up-regulated, and nine circRNAs were down-regulated (Figure 5F). To predict the potential function of the DE circRNAs, which associated host genes were subjected to GO and KEGG pathway analysis. The top 10 most enriched GO terms in each category are shown in Figure 5G, which serve as myeloid leukocyte activation in the BP category, cytoplasmic vesicle membrane in the CC category, and ICAM-3 receptor activity in the MF category. In addition, KEGG pathway analysis showed that DE circRNAs were enriched in several pathways, including HTLV-I infection, Epstein–Barr virus infection, and Viral myocarditis (Figure 5H).

**Figure 8:**
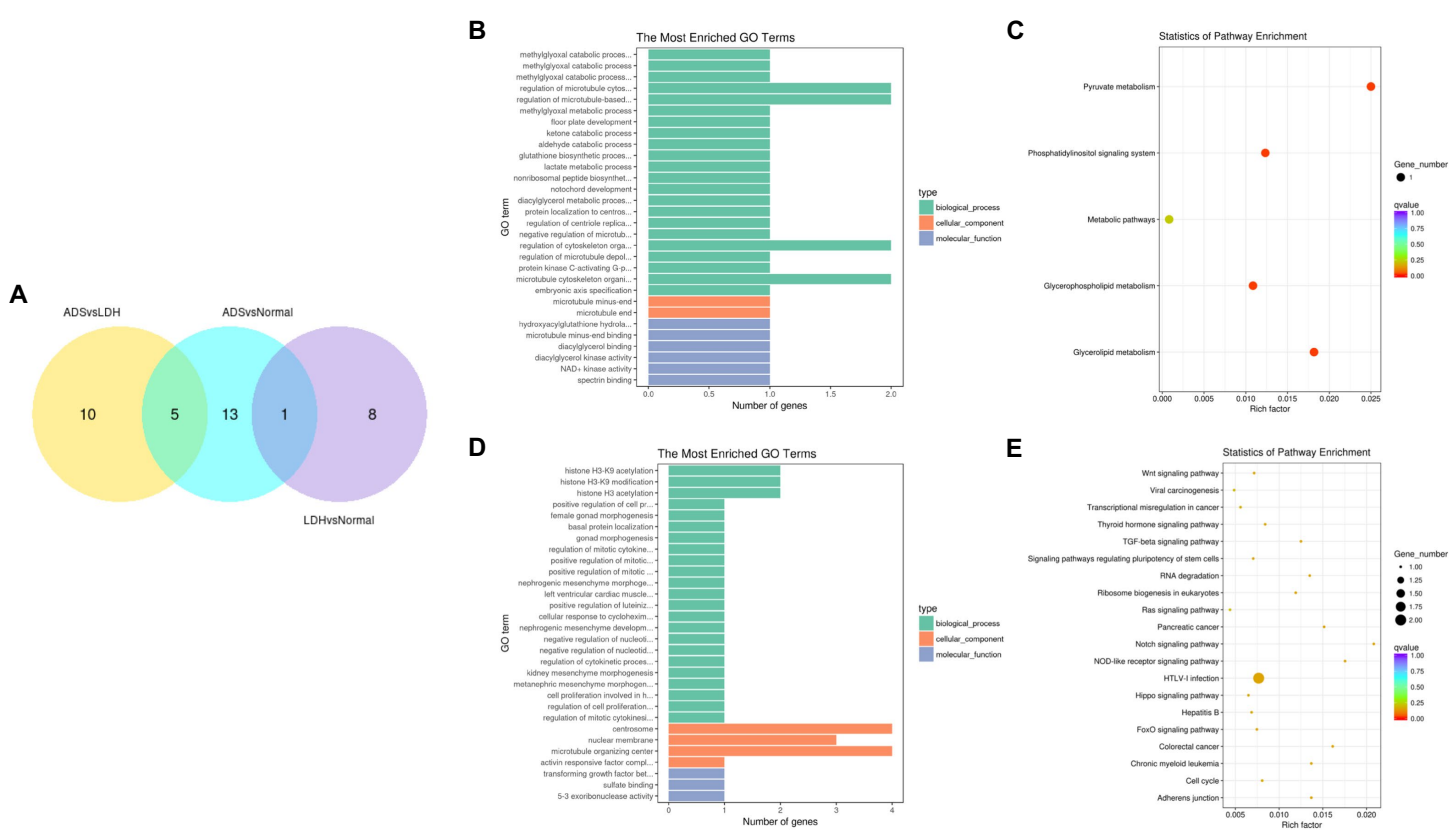
Identification of differentially expressed circRNAs **(A)** The Venn diagram created by the differentially expressed data of circRNAs. **(B)** The 20 most enriched Gene Ontology terms for the parental genes of 5 co-significantly DE circRNAs in the ADS compared to the LDH and Normal group. **(C)** The 20 most enriched KEGG pathways for 5 co-significantly DE circRNAs. **(D)** The 20 most enriched Gene Ontology terms for the parental genes of 10 significantly DE circRNAs in the ADS compared to the LDH group. **(E)** The 20 most enriched KEGG pathways for 10 significantly DE circRNAs. Enriched GO terms are on the vertical axis, and the number of annotated differentially expressed genes associated with each GO term is indicated on the horizontal axis. The size of the symbol represents the number of genes, and the colors represent *p*-value in KEGG.

In particular, overlapping analysis of the differential expression results from the three groups. As shown in Figure 8A, five circRNAs expressions were significantly different in the ADS compared to the LDH and Normal groups. The most abundant GO terms are shown in Figure 8B, which account for the regulation of cytoskeleton organization in the BP category, microtubule ends in the CC category, and NAD+ kinase activity in the MF category. In addition, KEGG pathway analysis revealed that DE circRNAs were enriched in several pathways, including Pyruvate metabolism, Glvcerolipid metabolism, Phosphatidylinositol signaling system, and Glycerophosoholipid metabolism (Figure 8C).

Interestingly, compared to the LDH group, only 10 circRNAs were independently present in ads. The corresponding GO terms are displayed in Figure 8D, of which the most enriched GO terms comprise histone H3-K9 acetylation in the BP category, microtubule organizing center in the CC category, and 5–3 exoribonuclease activity in the MF category. In addition, the KEGG pathway analysis indicated that DE circRNAs were enriched in several pathways, including HTLV-I infection, Notch signaling pathway, and NOD-like receptor signaling pathway (Figure 8E).

### The construction of lncRNA-mediated networks

**Figure 9:**
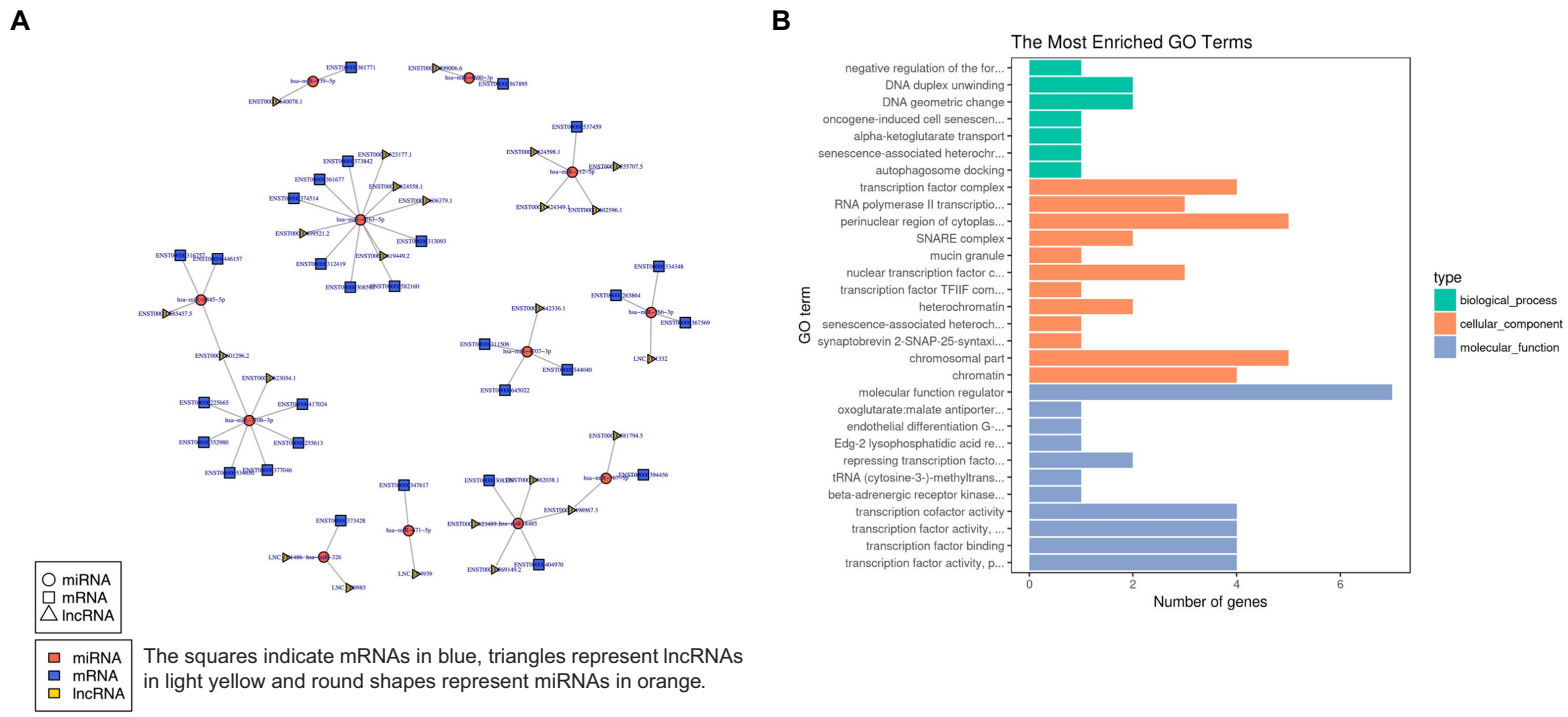
**(A)** lncRNA-miRNA-mRNA ceRNA network. **(B)** The 20 most enriched Gene Ontology terms for DEG in the network. Enriched GO terms are on the vertical axis, and the number of annotated differentially expressed genes associated with each GO term is indicated on the horizontal axis.

In order to characterize the underlying functions of lncRNAs, further exploring the potential connections between lncRNAs, miRNAs, and mRNAs, we constructed the lncRNA-miRNA-mRNA ceRNA network, which satisfied the |Pearson coefficient| > 0.9 and *p* < 0.05 (Figure 9A). Among them, a total of 65 junction nodes (including 24 lncRNAs, 12 miRNAs, and 29 mRNAs) were involved in the lncRNA-miRNA-mRNA ceRNA regulatory network. Actually, the network between lncRNAs, miRNAs, and mRNAs is complicated. The same miRNA can target different lncRNAs and mRNAs, while different miRNAs can target different lncRNAs and the same mRNAs. In addition, different lncRNA-miRNA-mRNA networks execute different biological functions depending on the ncRNAs involved, which could be candidates for subsequent functional analysis.

In addition, GO analysis was performed to assess the function of DEG in the network, which further investigates the key genes associated with ADS. The GO results reveal the most enriched GO terms comprise the BP category in DNA duplex unwinding and DNA geometric change, the CC category in the perinuclear region of cytoplasm, chromosomal part, etc., and MF class had significant enrichment in molecular function regulator (Figure 9B).

### RT-qPCR

**Figure 10:**
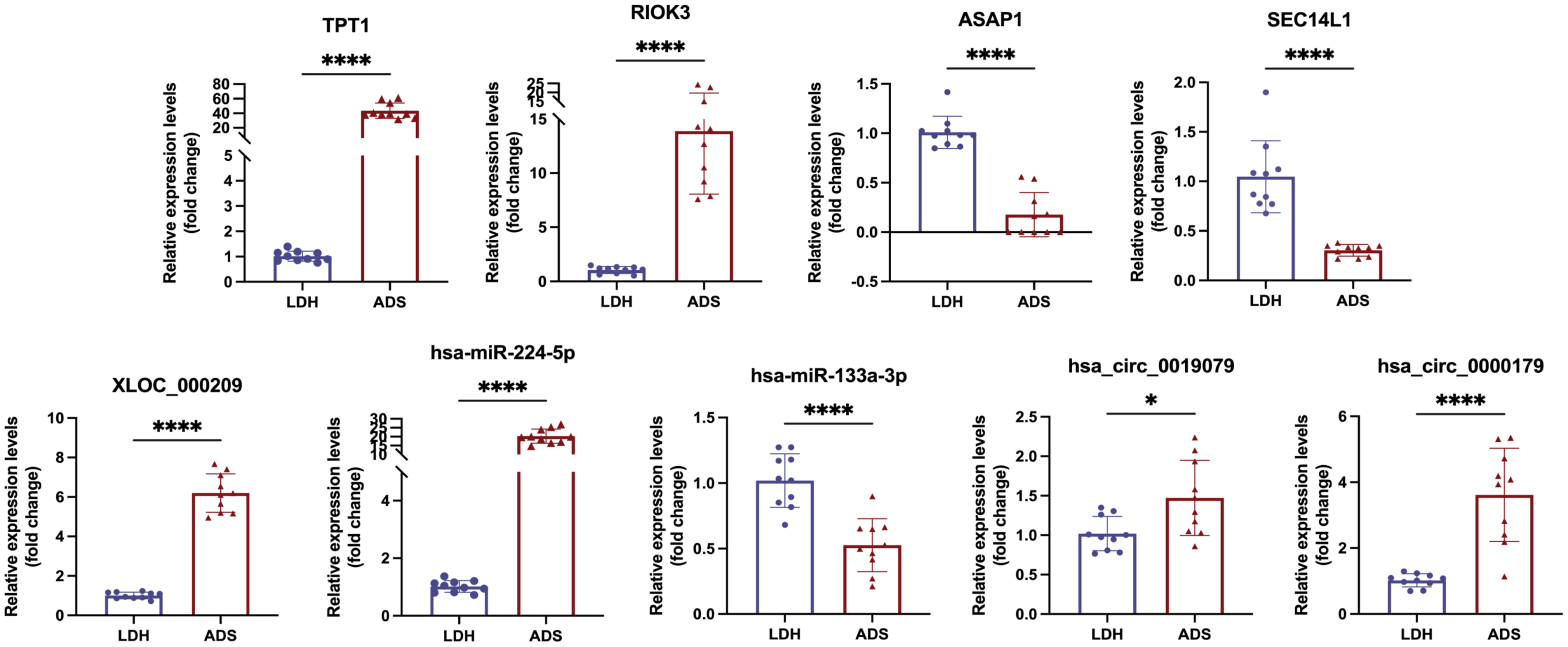
Validation for the expression of significant transcripts by quantitative RT-PCR (mean ± *SD*, *n* = 10; ****P<0.0001, *P<0.05 compared with the LDH group).

We focused on Top10 significantly differentially expressed RNAs in ADS vs. LDH. RT-qPCR was employed to detect the expression of DE RNAs in NP tissue samples (*n* = 10/group) from the ADS group and LDH group, respectively (Figure 10). Two up-regulated mRNAs (TPT1, RIOK3), two down-regulated mRNAs (ASAP1, SEC14L1), two up-regulated circRNAs (hsa_circ_0019079, hsa_circ_0000179), one up-regulated miRNA (hsa-miR-224-5p) and one down-regulated miRNA (hsa-miR-133a-3p) and the most significantly different lncRNA (XLOC_000209). Consequently, all these ncRNAs were confirmed to be significantly differentially expressed, consistent with the RNA sequencing results.

## Discussion

Typically, the pathogenesis of both ADS and LDH is considered to be based on disc degeneration, whereas the different disease endpoints are determined by the degree of asymmetric disc degeneration. Therefore, understanding the differential regulation of gene expression between ADS and LDH would be beneficial in developing potential biomarkers and predicting disease risk for ADS. Anatomically, the intervertebral disc consists of a gel-like core called the nucleus pulposus (NP), which is surrounded by a thin layer of fibrocartilage, called the annulus fibrosus (Yasuoka et al., 2007). NP cells play an essential role in maintaining the integrity of the disc by producing extracellular matrix (ECM) components, such as aggregated proteoglycans and type II and X collagen (Loreto et al., 2011). With age, disc fibrocartilage cells degenerate, and proteoglycan production decreases, leading to disc dehydration and collapse, with subsequent tearing and fracturing of the annulus fibrosus due to increased pressure, which ultimately induces NP herniation. Therefore, when the disc is subjected to repetitive mechanical stress, which could result in progressive, chronic disc herniation. Nowadays, growing evidence suggests that aberrant NP cell function is pivotal to the pathogenesis of IDD, including altered cell proliferation, apoptosis, ECM production/degradation, and cytokine secretion (Li et al., 2015). While genetic and environmental factors are influential in IDD, genetic factors appear to be the most significant (Guo et al., 2020). As is well-known, human serum or plasma is a consistently abundant source of biomarkers. Circulating RNA molecules could be analyzed to identify novel and promising biomarkers for numerous diseases (Li et al., 2014). Recent studies, using RNA-seq to assay the repertoire of genes within a tissue or cell have identified unique mechanistic pathways in human blood cells of patients with rheumatoid arthritis (Reynolds et al., 2013), IDD (Ma et al., 2022). In conclusion, taking advantage of cutting-edge techniques in RNA-seq analysis, we have unprecedentedly integrated mRNA, lncRNA, circRNA, and miRNA profiles in Normal, LDH, and ADS, analyzed the crosstalk of multiple ncRNAs in the regulation of disc degeneration while focusing on the ncRNAs associated with the pathogenesis of ADS.

Overall, overlapping the results of the three comparison groups (ADS vs. LDH, ADS vs. Normal, LDH vs. Normal), which involved 4 lncRNAs and 14 mRNAs. These co-expressed differentially ncRNAs are present throughout the process of intervertebral disc degeneration and play critical regulatory roles in LDH as well as ADS. The co-differentially expressed RNAs were enriched in the Rap1 signaling pathway, which is implicated in endocytosis and RNA transport throughout the process of disc degeneration. In detail, Rap1 is a micro-GTPase that controls a variety of processes, such as cell adhesion, cell–cell junction formation, and cell polarity. Rap1a and Rap1b, as two highly homologous micro-G proteins, are essential for *in vivo* angiogenesis and the normal EC response to vascular endothelial growth factor (VEGF). Rap1b interacts with VEGFR2 to manipulate angiogenesis *in vivo* (Lakshmikanthan et al., 2011).

Usually, normal IVD is predominantly an avascular structure, whereas blood vessels are merely present in the outer layers of the longitudinal ligaments and fibrous rings. The inward growth of vascularised granulation tissue occurs at the fissure of the torn disc, which is directly correlated with disc degeneration. The correlation between enhanced angiogenesis and the severity of degeneration could be estimated by extruding and isolating the optical density of the ECM (Koike et al., 2003). Several authors believe that neovascularisation is a response to injury, which could provide nutrition to the injured site of the disc (Virri et al., 1996). Alternative studies suggest that vascularisation of the annulus vasculosus is a response to trauma, where neovascularisation is associated with endothelial cells, fibroblasts, and monocytes (Pokharna and Phillips, 1998).

**Figure 11:**
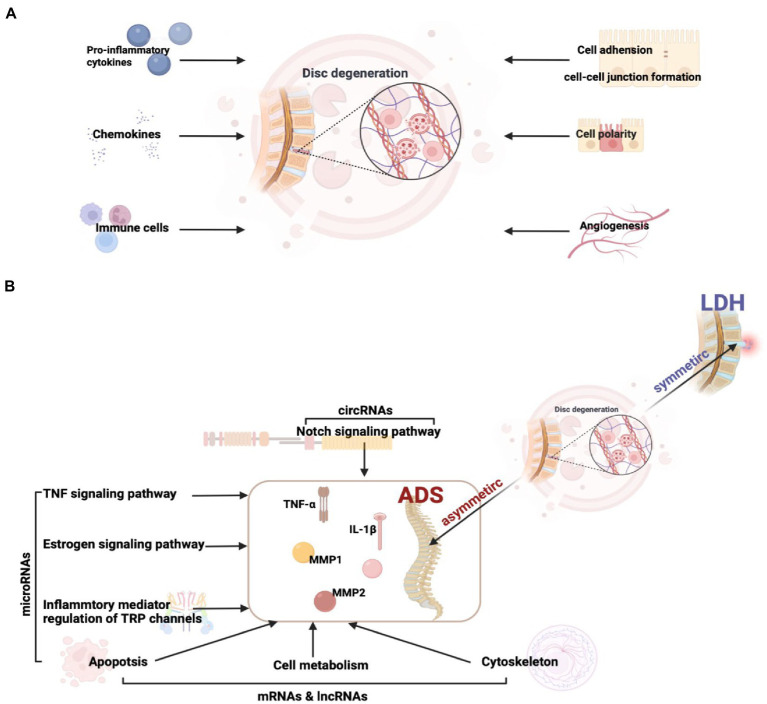
**(A)** Factors influencing intervertebral disc degeneration. **(B)** The key pathway and molecular mechanisms related to ADS from asymmetric disc degeneration.

The growth of vessels into the disc is multi-factorial, engaging the breakdown of the proteoglycan matrix of the IVD, the proliferation of granulation tissue, and the accumulation of cytokines and growth factors (Johnson et al., 2001; Kato et al., 2004; Johnson et al., 2005). The vascularised granulation tissue is clustered at the disc’s margins, indicating a hyper-intense accumulation of inflammatory factors intended to boost self-resorption. At the same time, endocytosis plays a vital role in normal cellular function by clearing foreign substances and protecting the host from pathogenic/viral attacks. Here, we conjecture that endocytosis is significantly enriched due to the intense feedback triggered in the organism by accumulating large amounts of inflammatory factors during disc degeneration (Figure 11A).

Intriguingly, although the pathological basis of both LDH and ADS involves disc degeneration, the mechanism of ADS-specific regulation remains elusive. In order to illuminate the crucial molecular mechanisms associated with the pathogenesis of ADS, our study focused on DE ncRNA between LDH and ADS. The results revealed that 99 lncRNAs, 10 circRNAs, 9 miRNAs, and 832 mRNAs were exclusively differentially expressed in the ADS group compared to LDH patients. In particular, miRNAs were significantly enriched in TNF signaling pathway, Inflammatory mediator regulation of TRP channels, Estrogen signaling pathway, and Apoptosis. Interestingly, in this study, the Notch signaling pathway, where DE circRNA (novel_circ_0004327, sourc_gene: ENSG00000114166, gene name: *KAT2B*) enrichment between ADS and LDH. lncRNA and mRNA are also enriched in pathways associated with cell metabolism, Apoptosis, and the cytoskeleton.

The narrowing of disc height, recognized as the most reliable radiological indication of disc degeneration, is typically characterized by a loss of ECM within the central NP region. Estrogen receptor β (ERβ) is expressed in human intervertebral discs (Gruber et al., 2002), which significantly decreases with aggravated disc degeneration (Song et al., 2014). Recently, evidence shows that estrogen could influence the severity of disc degeneration, the female rat intervertebral disc is prone to degeneration post oophorectomy (Wang et al., 2004). Hormone replacement therapy could maintain disc height in postmenopausal women (Baron et al., 2005). The intervertebral disc NP tissue is specialized hyaline cartilage. Interestingly, The interaction between estrogen and ERβ in chondrocytes could induce proteoglycan and collagen biosynthesis, which would maintain the homeostasis of the cartilage tissue (Richmond et al., 2000; Ham et al., 2004), as well as 17β-estradiol could suppress NPC apoptosis (Wang et al., 2021). Transient Receptor Potential (TRP) channels, which can constitute a family of multimodal ion channels, have recently emerged as contributors to disc pathology. In contrast to non-degenerative IVDs, TRPC6, TRPM2, and TRPML1 manifest enhanced expression of genes and proteins in degenerative IVDs (Sadowska et al., 2019).

Notch signaling pathway has been demonstrated to promote intervertebral disc cell proliferation (Hiyama et al., 2011). In rat NPC, the production of Notch1 and Notch2 receptors, ligands, and target genes was reinforced by TNF-α stimulation, while Notch2 levels were more abundant in degenerative IVD tissues versus non-degenerative tissues (Wang et al., 2013). In parallel, the JAG2/Notch2 axis could inhibit TNF-α-mediated apoptosis by blocking the composition of the RIP1-FADD-caspase-8 complex (Long et al., 2019). Long intergenic non-coding RNA-00917 regulates the proliferation, inflammation, and pyroptosis of NP cells *via* targeting miR-149-5p/NOD-like receptor protein 1 axis (Li et al., 2022).

The inflammatory process, exacerbated by TNF-α and IL-1β, is considered a key event during IDD (Risbud and Shapiro, 2014). The TNF-α expression is upregulated in IDD, which is tightly associated with various pathological processes, including inflammatory response, stromal destruction, cellular senescence, autophagy, apoptosis, cellular scorching, and proliferation (Wang et al., 2020). In dissociated human neural progenitor cells (hNPCs), the production of MMP-1, MMP-3, MMP-13, ADAMTS-4, and ADAMTS-5 is prominently stimulated by TNF-α, which contributes to the degradation of aggregated proteoglycans and collagen (Seguin et al., 2005). Besides, the JNK/ERK–MAPK and NF-κB signaling pathways in NPCs during IDD, modulated by TNF-α binding to TNF receptors, up-regulate pro-apoptotic and down-regulate anti-apoptotic proteins, thereby inducing apoptosis (Zhang et al., 2019). In NPCs, overexpression of miR-532-5p suppresses the activation of apoptosis-related proteins and the acceleration of apoptosis mediated by TNF-α, for instance, caspase-3, caspase-8, and caspase-9 (Zhu et al., 2020). Knockdown of miR-494 was found to protect NPC from TNF-α-mediated JunD-induced apoptosis *via* cytochrome C apoptotic signaling (Wang et al., 2015).

Moreover, we established the lncRNA-miRNA-mRNA ceRNA network to analyze the crosstalk of multiple ncRNAs in regulating ADS. In total, 65 nodes were defined in the network, including 24 lncRNAs, 12 miRNAs, and 29 mRNAs. Remarkably, microRNA-766-3p facilitates anti-inflammatory responses in human rheumatoid arthritis (RA) fibroblast-like synoviocyte MH7A cells *via* indirectly inhibiting NF-κB signaling (Hayakawa et al., 2019). The miR-326-5p dramatically enhances the angiogenic capacity of endothelial progenitor cells (EPCs; Li et al., 2019). The prevalence of angiogenesis was significantly correlated with microscopic calcifications in myeloid protrusion specimens (Karamouzian et al., 2010). At the molecular level, different proteins, collagen, and proteoglycan breakdown products probably induce angiogenesis (Thornalley, 1998), of which the strong expression of growth factors has been recognized as an essential regulator of neointima formation and disc degeneration (Peng et al., 2006).

At the cellular level, apoptosis is intimately interconnected with lumbar disc degeneration. For instance, in mature and aging discs, the apoptosis rate is significantly elevated in the NP tissue (Zeng et al., 2010). Type I collagen was detected in both the NP and the annulus fibrosus from normally and pathologically degenerated discs. In contrast, type II and IX collagen is merely increased in areas of mild degeneration but is even absent in more severely lesioned regions (Nerlich et al., 1997). As we know, the strength of the lumbar disc is related to the content of fluid and proteoglycans in the disc. The hydrophilicity of proteoglycans allows water introduction into the NP by osmosis, which compresses the disc’s interior. Unfortunately, the water content of proteoglycans and intervertebral discs tends to decrease with age (Antoniou et al., 1996). In line with previous studies, although the asymmetry of the intervertebral facet joints is a congenital structural feature, maintaining the age-related susceptibility in the sagittal or coronal plane, which could exacerbate the degree of facet joint asymmetry (Lai et al., 2018). Therefore, we hypothesize that DE ncRNA between LDH and ADS may be a potential genetic marker for ADS, whose mediated biological processes interact to be co-responsible for exacerbation of facet joint asymmetry, which consequently results in a more severe ADS disease endpoint, distinct from the common symmetric disc degeneration.

Overall, our study reveals potentially important RNAs, pathways, and RNA networks associated with ADS and provides a perspective view of differentially expressed RNAs’ potential functions and mechanisms. Simultaneously, this research provides a valuable RNA resource for future in-depth ADS investigations and establishes interaction networks, particularly the circRNA and miRNA could serve as emerging RNA molecules with unique criteria and clinical promises (Figure 11B).

However, the research still presented some flaws that deserve pondering and improvement. Firstly, based on sample size estimates, our RNA sequencing and bioinformatics analyses are anticipated to have more ADS samples, optimizing the study design for differential expression detection with more higher confidence level. Secondly, it ought to be noted that we performed RNA-seq from the peripheral blood of ADS patients rather than NP tissue due to the lack of samples in clinical practice, which implies a straightforward correlation between the analyzed RNA and ADS is necessary to be critically investigated in future studies. Finally, RNA regulatory networks are exclusively predicted based on bioinformatics, lacking validated practical experiments, which require future in-depth investigation through *in vivo* and *in vitro* experiments. The utilization of animal models is also a resourceful tool in ADS research, which would facilitate studying the potential functions and evolutionary conservation of RNA. Currently, our team is dedicated to investigating these potential functions and mechanisms.

## Conclusion

In summary, whole transcriptome sequencing revealed differential circRNA, lncRNA, miRNA, and mRNA expression profiles between whole blood samples, which were collected from healthy volunteers (non-degenerative changes), LDH patients (symmetric disc degeneration), and ADS patients (asymmetric disc degeneration). By exploring the differentially expressed transcriptome, GO term, and KEGG pathway analysis and regulatory network construction, we identified multiple ncRNAs that play key regulatory roles in the pathogenesis of ADS, including *miRNA-766-3p*, *miRNA-326*, *novel_circ_0004327*, *XLOC_000209*, *GRK2*. The discoveries have laid the foundation for further studies of non-coding RNAs associated with the development of ADS. However, further studies on larger cohorts of patients will be necessary to validate our conclusions.

## Data availability statement

The original contributions presented in the study are included in the article, further inquiries can be directed to the corresponding authors. The datasets presented in this study can be found in online repositories. The names of the repository/repositories and accession number(s) can be found at: www.ncbi.nlm.nih.gov/, GSE209825. The raw data can be found at https://www.jianguoyun.com/p/Ddm6koYQzI72ChjiwdcEIAA.

## Ethics statement

The studies involving human participants were reviewed and approved by Medical ethics committee of the Kunming Medical University Ethical. The patients/participants provided their written informed consent to participate in this study.

## Author contributions

PL, JS, and XS contributed to the conception and design of the study. XS and XW recruited and collected samples and conducted the experimental works. PL and XS performed the bioinformatic analysis and results visualization. XS and PL drafted and wrote the manuscript. All authors read, edited, and approved the final manuscript.

## Funding

This work was funded by the Key Academic Programs of Kunming Medical University in the 12th Five-Year Plan (grant number 0105008).

## Acknowledgments

We thank Dr. Zhihua Wang and Prof. Gang Zhao for their help with the sample collections. We thank Prof. Lanying Wang for constructive feedback on the manuscript. Figures 1 and 11 were created with BioRender.com.

## Conflict of interest

The authors declare that the research was conducted in the absence of any commercial or financial relationships that could be construed as a potential conflict of interest.

## Publisher’s note

All claims expressed in this article are solely those of the authors and do not necessarily represent those of their affiliated organizations, or those of the publisher, the editors and the reviewers. Any product that may be evaluated in this article, or claim that may be made by its manufacturer, is not guaranteed or endorsed by the publisher.

## Footnotes

^1^ http://rfam.sanger.ac.uk/

^2^ http://www.repeatmasker.org

^3^ http://www.mirbase.org

^4^ http://geneontology.org/

^5^ http://www.kegg.jp/

^6^ http://www.targetscan.org/

^7^ http://www.microrna.org/

## References

[ref1] Adams M. A. Roughley P. J. (2006). What is intervertebral disc degeneration, and what causes it? Spine 31, 2151–2161. doi: 10.1097/01.brs.0000231761.73859.2c 16915105

[ref2] Akhter R. (2018). Circular RNA and Alzheimer's disease. Adv. Exp. Med. Biol. 1087, 239–243. doi: 10.1007/978-981-13-1426-1_19 30259371

[ref3] Antoniou J. Steffen T. Nelson F. Winterbottom N. Hollander A. P. Poole R. A.. (1996). The human lumbar intervertebral disc: evidence for changes in the biosynthesis and denaturation of the extracellular matrix with growth, maturation, ageing, and degeneration. J. Clin. Invest. 98, 996–1003. doi: 10.1172/JCI118884, PMID: 8770872 PMC507515

[ref4] Baron Y. M. Brincat M. P. Galea R. Calleja N. (2005). Intervertebral disc height in treated and untreated overweight post-menopausal women. Hum. Reprod. 20, 3566–3570. doi: 10.1093/humrep/dei251, PMID: 16113041

[ref5] Beermann J. Piccoli M. T. Viereck J. Thum T. (2016). Non-coding RNAs in development and disease: background, mechanisms, and therapeutic approaches. Physiol. Rev. 96, 1297–1325. doi: 10.1152/physrev.00041.2015 27535639

[ref6] Benner B. Ehni G. (1979). Degenerative lumbar scoliosis. Spine (Phila Pa 1976) 4, 548–552. 515844

[ref7] Chen C. Tan H. N. Bi J. Q. Li Z. Rong T. H. Lin Y. X.. (2018). Identification of competing endogenous RNA regulatory networks in vitamin a deficiency-induced congenital scoliosis by Transcriptome sequencing analysis. Cell. Physiol. Biochem. 48, 2134–2146. doi: 10.1159/000492556, PMID: 30110682

[ref8] Chen X. Yang T. Wang W. Xi W. Zhang T. Li Q.. (2019). Circular RNAs in immune responses and immune diseases. Theranostics 9, 588–607. doi: 10.7150/thno.29678, PMID: 30809295 PMC6376182

[ref9] Correia de Sousa M. Gjorgjieva M. Dolicka D. Sobolewski C. Foti M. (2019). Deciphering miRNAs' action through miRNA editing. Int. J. Mol. Sci. 20:6249. doi: 10.3390/ijms20246249, PMID: 31835747 PMC6941098

[ref10] Cui S. Zhou Z. Liu X. Richards R. G. Alini M. Peng S.. (2020). Identification and characterization of serum microRNAs as biomarkers for human disc degeneration: an RNA sequencing analysis. Diagnostics 10:1063. doi: 10.3390/diagnostics10121063, PMID: 33302347 PMC7762572

[ref11] Gruber H. E. Yamaguchi D. Ingram J. Leslie K. Huang W. Miller T. A.. (2002). Expression and localization of estrogen receptor-beta in annulus cells of the human intervertebral disc and the mitogenic effect of 17-beta-estradiol in vitro. BMC Musculoskelet. Disord. 3:4. doi: 10.1186/1471-2474-3-4, PMID: 11846890 PMC65546

[ref12] Guo H.-Y. Guo M.-K. Wan Z.-Y. Song F. Wang H.-Q. (2020). Emerging evidence on noncoding-RNA regulatory machinery in intervertebral disc degeneration: a narrative review. Arthritis Res. Ther. 22:270. doi: 10.1186/s13075-020-02353-2, PMID: 33198793 PMC7667735

[ref13] Ham K. D. Oegema T. R. Loeser R. F. Carlson C. S. (2004). Effects of long-term estrogen replacement therapy on articular cartilage IGFBP-2, IGFBP-3, collagen and proteoglycan levels in ovariectomized cynomolgus monkeys. Osteoarthr. Cartil. 12, 160–168. doi: 10.1016/j.joca.2003.08.002, PMID: 14723875

[ref14] Haque S. Harries L. W. (2017). Circular RNAs (circRNAs) in health and disease. Genes (Basel) 8:353. doi: 10.3390/genes8120353, PMID: 29182528 PMC5748671

[ref15] Hayakawa K. Kawasaki M. Hirai T. Yoshida Y. Tsushima H. Fujishiro M.. (2019). MicroRNA-766-3p contributes to anti-inflammatory responses through the indirect inhibition of NF-κB signaling. Int. J. Mol. Sci. 20:809. doi: 10.3390/ijms20040809, PMID: 30769772 PMC6413049

[ref16] Herkowitz H. N. Kurz L. T. (1991). Degenerative lumbar spondylolisthesis with spinal stenosis. A prospective study comparing decompression with decompression and intertransverse process arthrodesis. J. Bone Joint Surg. Am. 73, 802–808. 2071615

[ref17] Hiyama A. Skubutyte R. Markova D. Anderson D. G. Yadla S. Sakai D.. (2011). Hypoxia activates the notch signaling pathway in cells of the intervertebral disc: implications in degenerative disc disease. Arthritis Rheum. 63, 1355–1364. doi: 10.1002/art.30246, PMID: 21305512 PMC3613279

[ref18] Hiyama A. Suyama K. Sakai D. Tanaka M. Watanabe M. (2022). Correlational analysis of chemokine and inflammatory cytokine expression in the intervertebral disc and blood in patients with lumbar disc disease. J. Orthop. Res. 40, 1213–1222. doi: 10.1002/jor.25136, PMID: 34191345

[ref19] Johnson W. E. B. Caterson B. Eisenstein S. M. Roberts S. (2005). Human intervertebral disc aggrecan inhibits endothelial cell adhesion and cell migration in vitro. Spine 30, 1139–1147. doi: 10.1097/01.brs.0000162624.95262.73, PMID: 15897827

[ref20] Johnson W. E. B. Eisenstein S. M. Roberts S. (2001). Cell cluster formation in degenerate lumbar intervertebral discs is associated with increased disc cell proliferation. Connect. Tissue Res. 42, 197–207. doi: 10.3109/03008200109005650, PMID: 11913491

[ref21] Karali M. Banfi S. (2019). Non-coding RNAs in retinal development and function. Hum. Genet. 138, 957–971. doi: 10.1007/s00439-018-1931-y 30187163

[ref22] Karamouzian S. Eskandary H. Faramarzee M. Saba M. Safizade H. Ghadipasha M.. (2010). Frequency of lumbar intervertebral disc calcification and angiogenesis, and their correlation with clinical, surgical, and magnetic resonance imaging findings. Spine 35, 881–886. doi: 10.1097/BRS.0b013e3181b9c986, PMID: 20354479

[ref23] Kato T. Haro H. Komori H. Shinomiya K. (2004). Sequential dynamics of inflammatory cytokine, angiogenesis inducing factor and matrix degrading enzymes during spontaneous resorption of the herniated disc. J. Orthop. Res. 22, 895–900. doi: 10.1016/j.orthres.2003.11.008, PMID: 15183452

[ref24] Koike Y. Uzuki M. Kokubun S. Sawai T. (2003). Angiogenesis and inflammatory cell infiltration in lumbar disc herniation. Spine 28, 1928–1933. doi: 10.1097/01.Brs.0000083324.65405.Ae, PMID: 12973136

[ref25] Kristensen L. S. Andersen M. S. Stagsted L. V. W. Ebbesen K. K. Hansen T. B. Kjems J. (2019). The biogenesis, biology and characterization of circular RNAs. Nat. Rev. Genet. 20, 675–691. doi: 10.1038/s41576-019-0158-7 31395983

[ref26] Lai Q. Liu Y. Guo R. Lv X. Wang Q. Zhu J.. (2018). A study of lumbar disc herniation and facet joint asymmetry. Int. Surg. 103, 87–94. doi: 10.9738/intsurg-d-16-00119.1

[ref27] Lakshmikanthan S. Sobczak M. Chun C. Henschel A. Dargatz J. Ramchandran R.. (2011). Rap1 promotes VEGFR2 activation and angiogenesis by a mechanism involving integrin αvβ₃. Blood 118, 2015–2026. doi: 10.1182/blood-2011-04-349282, PMID: 21636859 PMC3158727

[ref28] Li T. Peng Y. Chen Y. Huang X. Li X. Zhang Z.. (2022). Long intergenic non-coding RNA −00917 regulates the proliferation, inflammation, and pyroptosis of nucleus pulposus cells via targeting miR-149-5p/NOD-like receptor protein 1 axis. Bioengineered 13, 6036–6047. doi: 10.1080/21655979.2022.2043100, PMID: 35184666 PMC8974084

[ref29] Li X. Xue X. Sun Y. Chen L. Zhao T. Yang W.. (2019). MicroRNA-326-5p enhances therapeutic potential of endothelial progenitor cells for myocardial infarction. Stem Cell Res Ther 10:323. doi: 10.1186/s13287-019-1413-8, PMID: 31730013 PMC6858781

[ref30] Li Z. Yu X. Shen J. X. Chan M. T. V. Wu W. K. K. (2015). MicroRNA in intervertebral disc degeneration. Cell Prolif. 48, 278–283. doi: 10.1111/cpr.12180, PMID: 25736871 PMC6496566

[ref31] Li M. Zeringer E. Barta T. Schageman J. Cheng A. Vlassov A. V. (2014). Analysis of the RNA content of the exosomes derived from blood serum and urine and its potential as biomarkers. Philos. Trans. R. Soc. Lond. Ser. B Biol. Sci. 369:20130502. doi: 10.1098/rstb.2013.0502, PMID: 25135963 PMC4142023

[ref32] Long J. Wang X. du X. Pan H. Wang J. Li Z.. (2019). JAG2/Notch2 inhibits intervertebral disc degeneration by modulating cell proliferation, apoptosis, and extracellular matrix. Arthritis Res. Ther. 21:213. doi: 10.1186/s13075-019-1990-z, PMID: 31619270 PMC6796488

[ref33] Loreto C. Musumeci G. Castorina A. Loreto C. Martinez G. (2011). Degenerative disc disease of herniated intervertebral discs is associated with extracellular matrix remodeling, vimentin-positive cells and cell death. Ann. Anat. 193, 156–162. doi: 10.1016/j.aanat.2010.12.001, PMID: 21330123

[ref01] Ma X. Su J. Wang B. Jin X. (2022). Identification of Characteristic Genes in Whole Blood of Intervertebral Disc Degeneration Patients by Weighted Gene Coexpression Network Analysis (WGCNA). Comput. Math Methods Med. 2022:6609901. doi: 10.1155/2022/6609901 35069789 PMC8776439

[ref34] Mayer J. E. Iatridis J. C. Chan D. Qureshi S. A. Gottesman O. Hecht A. C. (2013). Genetic polymorphisms associated with intervertebral disc degeneration. Spine J. 13, 299–317. doi: 10.1016/j.spinee.2013.01.041, PMID: 23537453 PMC3655694

[ref35] Nerlich A. G. Schleicher E. D. Boos N. (1997). 1997 Volvo award winner in basic science studies. Immunohistologic markers for age-related changes of human lumbar intervertebral discs. Spine (Phila Pa 1976) 22, 2781–2795. doi: 10.1097/00007632-199712150-00001, PMID: 9431614

[ref36] Patop I. L. Wust S. Kadener S. (2019). Past, present, and future of circRNAs. EMBO J. 38:e100836. doi: 10.15252/embj.2018100836, PMID: 31343080 PMC6694216

[ref37] Peng B. G. Hao J. H. Hou S. X. Wu W. W. Jiang D. Y. Fu X. B.. (2006). Possible pathogenesis of painful intervertebral disc degeneration. Spine 31, 560–566. doi: 10.1097/01.brs.0000201324.45537.46 16508552

[ref38] Ploumis A. Transfledt E. E. Denis F. (2007). Degenerative lumbar scoliosis associated with spinal stenosis. Spine J. 7, 428–436. doi: 10.1016/j.spinee.2006.07.015 17630141

[ref39] Pokharna H. K. Phillips F. M. (1998). Collagen crosslinks in human lumbar intervertebral disc aging. Spine 23, 1645–1648. doi: 10.1097/00007632-199808010-00005, PMID: 9704370

[ref40] Reh T. A. Hindges R. (2018). MicroRNAs in retinal development. Annu. Rev. Vis. Sci. 4, 25–44. doi: 10.1146/annurev-vision-091517-034357 29889656

[ref41] Reynolds R. J. Cui X. Vaughan L. K. Redden D. T. Causey Z. Perkins E.. (2013). Gene expression patterns in peripheral blood cells associated with radiographic severity in African Americans with early rheumatoid arthritis. Rheumatol. Int. 33, 129–137. doi: 10.1007/s00296-011-2355-3, PMID: 22238028 PMC3769702

[ref42] Richmond R. S. Carlson C. S. Register T. C. Shanker G. Loeser R. F. (2000). Functional estrogen receptors in adult articular cartilage: estrogen replacement therapy increases chondrocyte synthesis of proteoglycans and insulin-like growth factor binding protein 2. Arthritis Rheum. 43, 2081–2090. doi: 10.1002/1529-0131(200009)43:9<2081::AID-ANR20>3.0.CO;2-I, PMID: 11014360

[ref43] Risbud M. V. Shapiro I. M. (2014). Role of cytokines in intervertebral disc degeneration: pain and disc content. Nat. Rev. Rheumatol. 10, 44–56. doi: 10.1038/nrrheum.2013.160, PMID: 24166242 PMC4151534

[ref44] Rong D. Sun H. Li Z. Liu S. Dong C. Fu K.. (2017). An emerging function of circRNA-miRNAs-mRNA axis in human diseases. Oncotarget 8, 73271–73281. doi: 10.18632/oncotarget.19154, PMID: 29069868 PMC5641211

[ref45] Sadowska A. Hitzl W. Karol A. Jaszczuk P. Cherif H. Haglund L.. (2019). Differential regulation of TRP channel gene and protein expression by intervertebral disc degeneration and back pain. Sci. Rep. 9:18889. doi: 10.1038/s41598-019-55212-9, PMID: 31827137 PMC6906425

[ref46] Schwab F. Dubey A. Gamez L. El Fegoun A. B. Hwang K. Pagala M.. (2005). Adult scoliosis: prevalence, SF-36, and nutritional parameters in an elderly volunteer population. Spine (Phila Pa 1976) 30, 1082–1085. doi: 10.1097/01.brs.0000160842.43482.cd, PMID: 15864163

[ref47] Seguin C. A. Pilliar R. M. Roughley P. J. Kandel R. A. (2005). Tumor necrosis factor-alpha modulates matrix production and catabolism in nucleus pulposus tissue. Spine (Phila Pa 1976) 30, 1940–1948. doi: 10.1097/01.brs.0000176188.40263.f9, PMID: 16135983

[ref48] Shi X. Li P. Wu X. Wang Z. Zhao G. Shu J. (2022). RNA-Seq comprehensive analysis reveals the Long noncoding RNA expression profile and Coexpressed mRNA in adult degenerative scoliosis. Front. Genet. 13:902943. doi: 10.3389/fgene.2022.902943, PMID: 36035195 PMC9403536

[ref49] Song X. X. Yu Y. J. Li X. F. Liu Z. D. Yu B. W. Guo Z. (2014). Estrogen receptor expression in lumbar intervertebral disc of the elderly: gender- and degeneration degree-related variations. Joint Bone Spine 81, 250–253. doi: 10.1016/j.jbspin.2013.09.002, PMID: 24838202

[ref50] Tay Y. Rinn J. Pandolfi P. P. (2014). The multilayered complexity of ceRNA crosstalk and competition. Nature 505, 344–352. doi: 10.1038/nature12986, PMID: 24429633 PMC4113481

[ref51] Thornalley P. J. (1998). Cell activation by glycated proteins. AGE receptors, receptor recognition factors and functional classification of AGEs. Cell. Mol. Biol. 44, 1013–1023. 9846883

[ref52] Virri J. Gronblad M. Savikko J. Palmgren T. Seitsalo S. Ruuskanen M.. (1996). Prevalence, morphology, and topography of blood vessels in herniated disc tissue. A comparative immunocytochemical study. Spine (Phila Pa 1976) 21, 1856–1863. doi: 10.1097/00007632-199608150-00004, PMID: 8875716

[ref53] Wang Y. Che M. Xin J. Zheng Z. Li J. Zhang S. (2020). The role of IL-1β and TNF-α in intervertebral disc degeneration. Biomed. Pharmacother. 131:110660. doi: 10.1016/j.biopha.2020.110660 32853910

[ref54] Wang H. Li Z. Huo Y. Tian T. Yang D. Ma L.. (2021). 17β-estradiol alleviates intervertebral disc degeneration by inhibiting NF-κB signal pathway. Life Sci. 284:119874. doi: 10.1016/j.lfs.2021.119874, PMID: 34390725

[ref55] Wang T. Li P. Ma X. Tian P. Han C. Zang J.. (2015). MicroRNA-494 inhibition protects nucleus pulposus cells from TNF-alpha-induced apoptosis by targeting JunD. Biochimie 115, 1–7. doi: 10.1016/j.biochi.2015.04.011, PMID: 25906693

[ref56] Wang H. Tian Y. Wang J. Phillips K. L. E. Binch A. L. A. Dunn S.. (2013). Inflammatory cytokines induce NOTCH signaling in nucleus pulposus cells: implications in intervertebral disc degeneration. J. Biol. Chem. 288, 16761–16774. doi: 10.1074/jbc.M112.446633, PMID: 23589286 PMC3675609

[ref57] Wang T. Zhang L. Huang C. Cheng A. G. Dang G. T. (2004). Relationship between osteopenia and lumbar intervertebral disc degeneration in ovariectomized rats. Calcif. Tissue Int. 75, 205–213. doi: 10.1007/s00223-004-0240-8, PMID: 15185057

[ref58] Yasuoka H. Asazuma T. Nakanishi K. Yoshihara Y. Sugihara A. Tomiya M.. (2007). Effects of reloading after simulated microgravity on proteoglycan metabolism in the nucleus pulposus and anulus fibrosus of the lumbar intervertebral disc. Spine 32, E734–E740. doi: 10.1097/BRS.0b013e31815b7e51, PMID: 18245988

[ref59] Zeng S. Liu L. Wang J. (2010). Significance of BNIP3 gene expression and cell apoptosis in nucleus pulposus of degenerative intervertebral disc in rabbits. Zhongguo Xiu Fu Chong Jian Wai Ke Za Zhi 24, 1367–1371. 21226364

[ref60] Zhang J. Wang X. Liu H. Li Z. Chen F. Wang H.. (2019). TNF-alpha enhances apoptosis by promoting chop expression in nucleus pulposus cells: role of the MAPK and NF-kappaB pathways. J. Orthop. Res. 37, 697–705. doi: 10.1002/jor.24204, PMID: 30561076

[ref61] Zhu G. Yang X. Peng C. Yu L. Hao Y. (2020). Exosomal miR-532-5p from bone marrow mesenchymal stem cells reduce intervertebral disc degeneration by targeting RASSF5. Exp. Cell Res. 393:112109. doi: 10.1016/j.yexcr.2020.112109, PMID: 32464126

[ref62] Zhu J. Zhang X. Gao W. Hu H. Wang X. Hao D. (2019). lncRNA/circRNAmiRNAmRNA ceRNA network in lumbar intervertebral disc degeneration. Mol. Med. Rep. 20, 3160–3174. doi: 10.3892/mmr.2019.10569, PMID: 31432173 PMC6755180

